# Identification of genomic regions associated with multi-silique trait in *Brassica napus*

**DOI:** 10.1186/s12864-019-5675-4

**Published:** 2019-04-23

**Authors:** Liang Chai, Jinfang Zhang, Kun Lu, Haojie Li, Lintao Wu, Hongshen Wan, Benchuan Zheng, Cheng Cui, Jun Jiang, Liangcai Jiang

**Affiliations:** 1 Crop Research Institute, Sichuan Academy of Agricultural Sciences, Chengdu, 610066 Sichuan China; 2 College of Agronomy and Biotechnology, Southwest University, Chongqing, 400716 Beibei China; 3 College of Life Science, Yangtze University, Jingzhou, 434025 Hubei China; 4 Rape Research Institute, Guizhou Academy of Agricultural Sciences, Guiyang, 550008 China

**Keywords:** Association analysis, *Brassica napus* L., Multi-silique, Near-isogenic line, Transcriptome sequencing, Whole genome re-sequencing

## Abstract

**Background:**

Although rapeseed (*Brassica napus* L.) mutant forming multiple siliques was morphologically described and considered to increase the silique number per plant, an important agronomic trait in this crop, the molecular mechanism underlying this beneficial trait remains unclear. Here, we combined bulked-segregant analysis (BSA) and whole genome re-sequencing (WGR) to map the genomic regions responsible for the multi-silique trait using two pools of DNA from the near-isogenic lines (NILs) zws-ms (multi-silique) and zws-217 (single-silique). We used the Euclidean Distance (ED) to identify genomic regions associated with this trait based on both SNPs and InDels. We also conducted transcriptome sequencing to identify differentially expressed genes (DEGs) between zws-ms and zws-217.

**Results:**

Genetic analysis using the ED algorithm identified three SNP- and two InDel-associated regions for the multi-silique trait. Two highly overlapped parts of the SNP- and InDel-associated regions were identified as important intersecting regions, which are located on chromosomes A09 and C08, respectively, including 2044 genes in 10.20-MB length totally. Transcriptome sequencing revealed 129 DEGs between zws-ms and zws-217 in buds, including 39 DEGs located in the two abovementioned associated regions. We identified candidate genes involved in multi-silique formation in rapeseed based on the results of functional annotation.

**Conclusions:**

This study identified the genomic regions and candidate genes related to the multi-silique trait in rapeseed.

**Electronic supplementary material:**

The online version of this article (10.1186/s12864-019-5675-4) contains supplementary material, which is available to authorized users.

## Background

In flowering plants, the pistil develops into a fruit, a process crucial for both yield and seed quality. Many recent studies have focused on abnormal pistil development, such as the formation of pistillodes (sterile pistils), pistil number variation, and mutations affecting pistil length [1–6]. Of these, pistil number variation, or the multi-pistil phenomenon, has also been reported in other plants, such as alfalfa (*Medicago sativa*) [1, 7], wheat (*Triticum aestivum*) [8, 9], sweet cherry (*Prunus avium*) [10], and rye (*Secale cereale*) [11].

*Brassica* crops are important sources of edible oil, vegetables, industrial erucic acid, animal feed (seed meal), and ornamental plants. Like most Brassicaceae family members, *Brassica* species have monoclinous flowers, with one pistil and six stamens per normal flower.

Morphological variation in siliques (pods) has been reported in several *Brassica* species. In the 1940s, Pathak [12] reported an abnormal plant of *Brassica campestris* L. var. sarson prain, which had one to two extra pods telescoped into the outermost pods. The inner pods was basically normal but were contorted and smaller than those of the wild type. Multi-locular *Brassica juncea* [13], trilocular *B. juncea* [14, 15] and *Brassica rapa* [16] were obtained from interspecific hybridization. Unlike previously reported fruit variations, zws-ms flower has three completely independent and apocarpous pistils and nine to ten stamens sharing the same receptacle in each floral organ, resulting in the formation of three independent siliques sharing the same carpopodium. Thus, zws-ms has an increased number of pod on each carpopodium rather than an increased number of loculi per pod. Similar phenotypes have also been observed previously: Guan and Li [17] obtained a line with double siliques through treatment of *B. napus* with ^12^C heavy ion beam irradiation. Hu et al. [18] identified a multi-pistil plant in a *B. campestris* L. cytoplasmic male sterile line, different from our zws-ms plants which were obtained by crossing of *B. napus* and *B. rapa* [19]. Though the agronomic and physiological traits, and genetic mechanism of zws-ms plants have been analyzed, the molecular mechanism underlying multi-pistil formation, which increased the silique number per plant in rapeseed, remains elusive.

Bulked segregant analysis (BSA) [20] is a simple but effective strategy for identifying molecular markers linked to target genes by genotyping a pair of bulked DNA samples from two populations with distinct phenotypes. BSA was usually combined with traditional markers, such as simple sequence repeat (SSR) [21], sequence related amplified polymorphism (SRAP) [22] or random amplified polymorphic DNA (RAPD) [23] markers to map traits. Recent years, combination of BSA and next-generation sequencing (NGS) have been successfully used in the identification of key genes/loci related to regular agronomic traits in *B. napus*. For example, Wang et al. combined BSA with NGS to map quantitative trait loci (QTL) for the branch angle and revealed *BnaYUCCA6* was candidate gene controlling branch angle [24]. Geng et al. combined specific length amplified fragment sequencing (SLAF-seq) with BSA and identified four candidate genes related to seed weight [25]. Yao et al. mapped the orange petal color gene (*Bnpc1*) in spring *B. napus* to a 151-kb region via BSA with whole genome re-sequencing (WGR) [26]. Hence, we believed that combining BSA and WGR would be a convenient way to map the genomic regions responsible for the multi-silique trait.

In this study, we performed association analysis based on SNP and InDel data using a Euclidean Distance (ED) algorithm to identify the gene underlying the multi-silique trait in rapeseed. Though the SNP-index method is also a classical method for association region identification, and has been employed in many studies [25, 26]. However, it is suitable in the case when four DNA samples are sequenced: two parental plants and two extreme pools from segregated population. In this study, only two pools from NILs multi-silique pool and single-silique pool were sequenced, therefore, only ED algorithm was useful in this situation. We also performed transcriptome sequencing to identify differentially expressed genes (DEGs). Based on functional annotation of the genes with SNPs or InDels and DEGs in the associated regions, we identified candidate genes that might be closely related to the multi-silique trait.

## Results

### Morphological analysis of the multi-silique trait in zws-ms

**Fig. 1:**
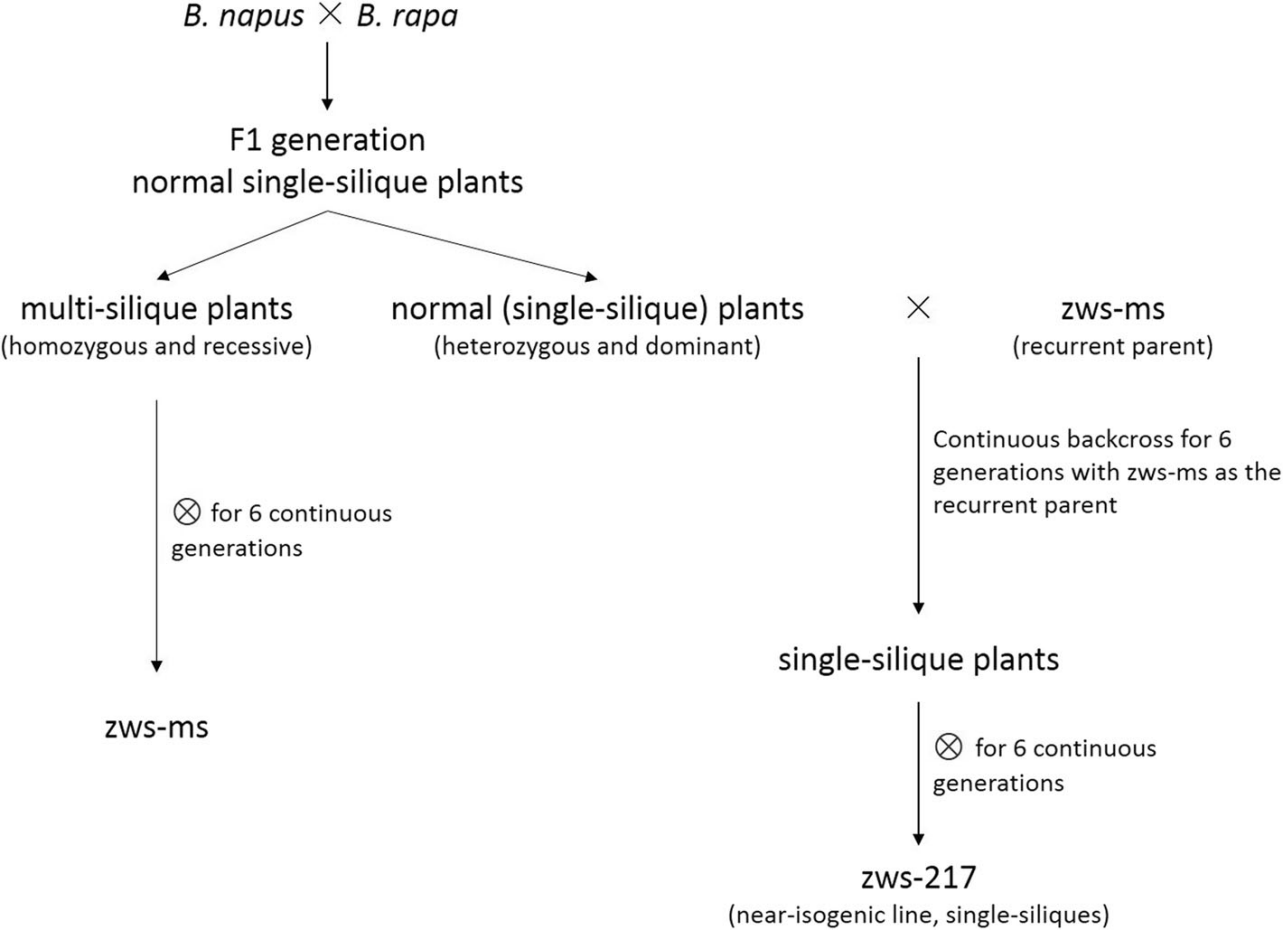
Scheme for constructing the multi-silique population (zws-ms) and the corresponding single-silique NIL population (zws-217)

**Fig. 2:**
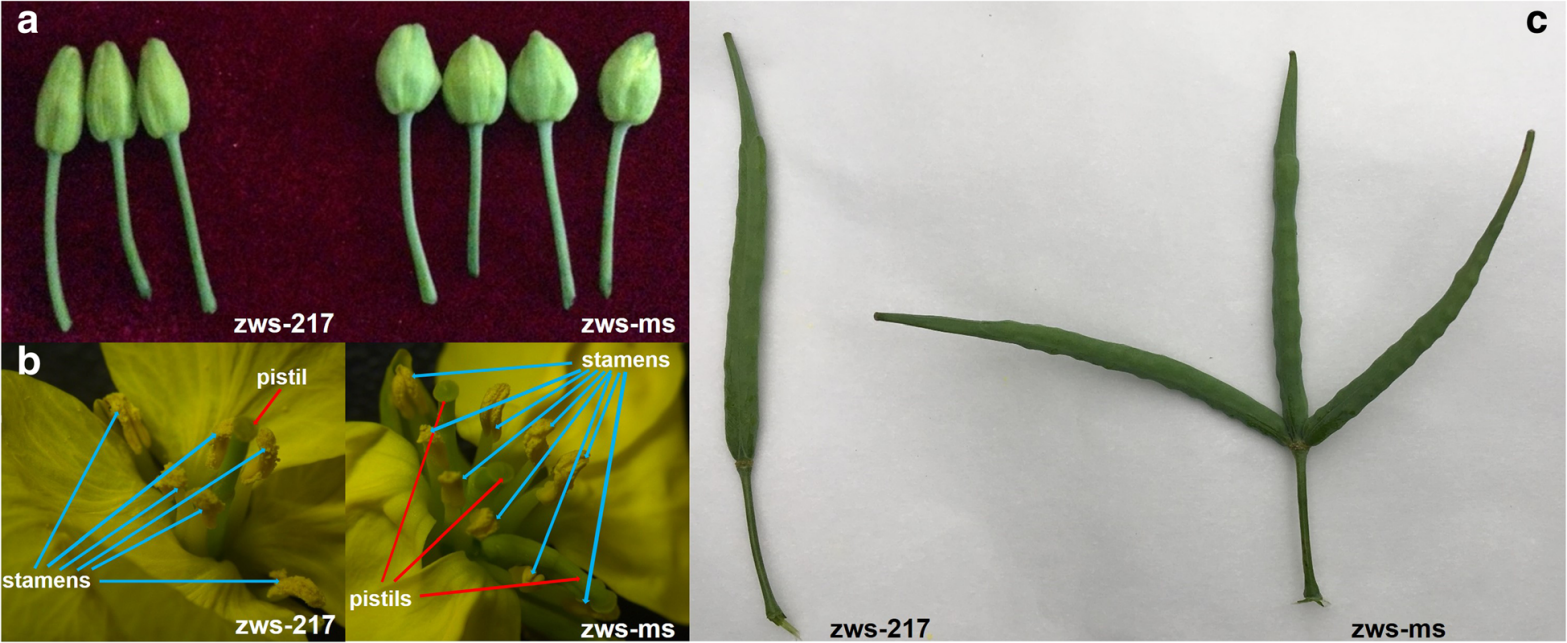
Morphological differences between zws-217and zws-ms. **a**: at the budding stage, zws-ms has inflated, round buds; **b**: at the full-bloom stage, a normal flower has one pistil and six stamens, while zws-ms has three pistils and nine stamens per floral organ; **c**: a normal silique in zws-217 and the three-silique trait in zws-ms. These images were taken originally in 2017

Under normal growth conditions, the NILs (Fig. 1) zws-ms (multi-silique) and zws-217 (single silique) showed no phenotypic differences (e.g., plant height, leaf shape, and number of branches) at the vegetative stage. However, zws-ms had shorter and rounder buds than zws-217 (Fig. 2a). At the full-bloom stage, zws-ms showed distinct floral structures with zws-217. In contrast to normal Brassicaceae flowers, which typically contain a single pistil and six stamens (four tetradynamous stamens and two shorter stamens), zws-ms flowers had three completely independent apocarpous pistils sharing the same receptacle in each floral organ (Fig. 2b). The zws-ms flower had nine to ten stamens, six of which were more prominent than the others (Fig. 2b). In zws-ms flowers, all pistils and stamens shared the same receptacle, but the petals and calyxes appeared normal. Roughly 32~53% of flowers in a zws-ms plant had this mutant multi-pistil and stamen configuration, whereas the rest flowers in the same plant were normal. The abnormal zws-ms flowers developed into three independent siliques sharing the same carpopodium as the flowers (Fig. 2c). The triple siliques were normal in length, but occasionally one became degenerated and curved.

### Association analysis

High-throughput whole genome re-sequencing on the Illumina HiSeq platform produced a total of 89.56 Gb of raw data. The Q30 ratio averaged 90.52%. After alignment to the reference genome, the average coverage was 91.78% and the average sequencing depth was 40×. When the clean reads were mapped to the *B. napus* reference genome, each of them was aligned in both forward and reverse directions; thus, the number of total reads was twice of the clean reads: zws-ms and zws-217 contained 328,507,566 and 263,588,948 reads, with 89.89 and 89.10% properly mapped to the reference genome, respectively.

**Fig. 3:**
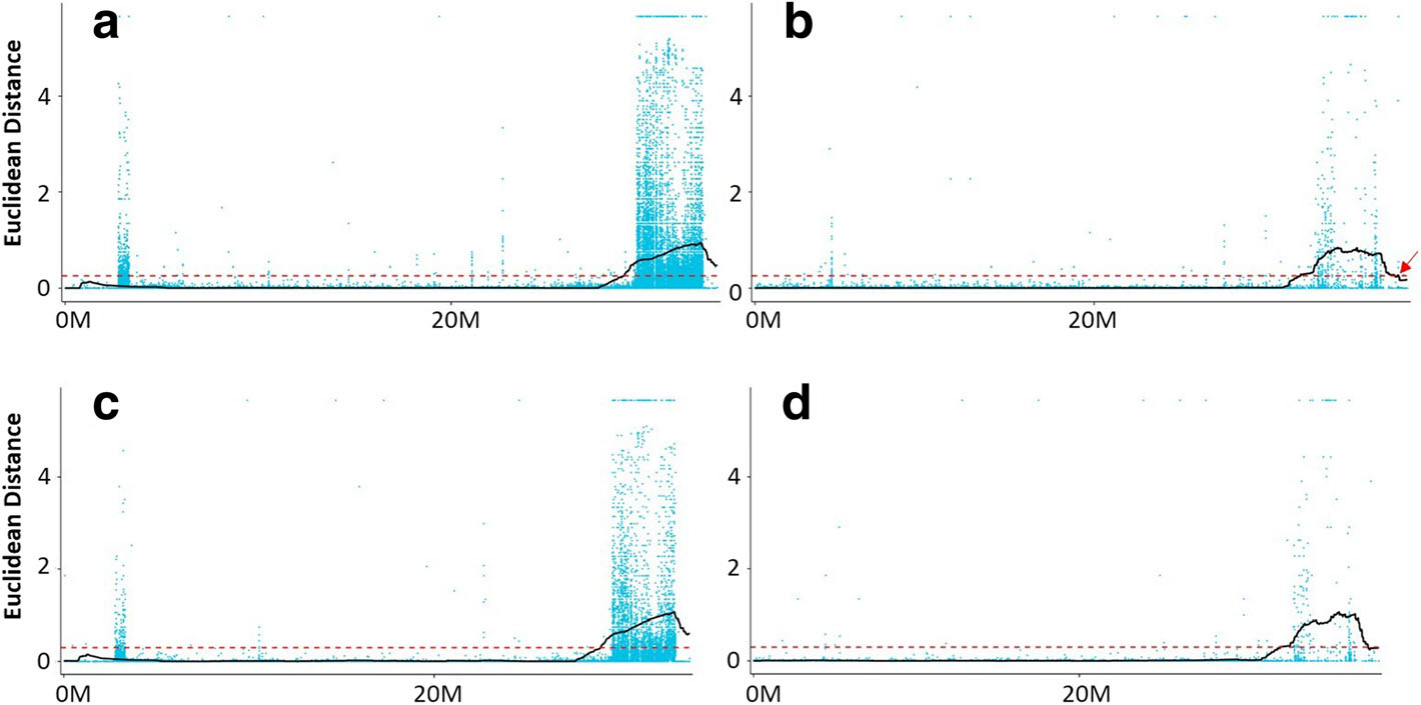
Associated regions calculated using the ED algorithm. Black solid lines represent the fitted values; red dotted lines represent the associated threshold. **a**: SNP-associated region on ChrA09; **b**: two SNP-associated regions on ChrC08; the smaller region is indicated by a red arrow; **c**: InDel-associated region on ChrA09; **d**: InDel-associated region on ChrC08

After filtration, 1,135,690 SNPs and 289,832 small InDels of high quality (HQ) were used for association analysis. We used the ED algorithm due to only two pools from NILs zws-ms and zws-217 were sequenced. According to the ED algorithm [27], the association threshold was 0.26. Based on this information, three SNP-associated regions were identified, including one on chromosome A09 (Fig. 3a) and two on chromosome C08 (Fig. 3b). One smaller associated region on the chromosome C08 that close to another larger one (Fig. 3b) was also detected. These regions had a size of 10.45 MB and contained 2095 genes. Similarly, two InDel-associated regions were identified on chromosomes A09 (Fig. 3c) and C08 (Fig. 3d) totaling 10.20 MB and containing 2044 genes, with an association threshold value of 0.30. Combining the SNP-associated regions and InDel-associated regions, two merged intersecting regions (Table 1, Additional file 1: Figure S1) were obtained on chromosomes A09 and C08, with 2044 genes in this 10.20 MB region, including 126 genes with non-synonymous mutations and 23 with frame-shift mutations.

**Table 1:** Information about the two intersecting regions

Chromosome	Start position	End position	Size (Mb)	Number of genes
A09	28,990,000	33,790,000	4.8	998
C08	32,340,000	37,740,000	5.4	1046
Total	–	–	10.2	2044

### Transcriptome sequencing, data mapping, and annotation

We performed sequencing saturation and cluster analysis of the samples to ensure the validity of the data. In total, 61 Gb of raw data were generated, with an average Q30 value of 90.54%. For each sample, approximately 36.7 M clean reads were generated, with an average GC content of 47.23% (Additional file 2: Table S1). The average proportion of total reads mapped to the reference genome of samples T01 to T06 was 73.729% (Additional file 3: Table S2), ranging from 77.81 to 76.03%.

### Annotation of genes containing SNPs and InDels

**Fig. 4:**
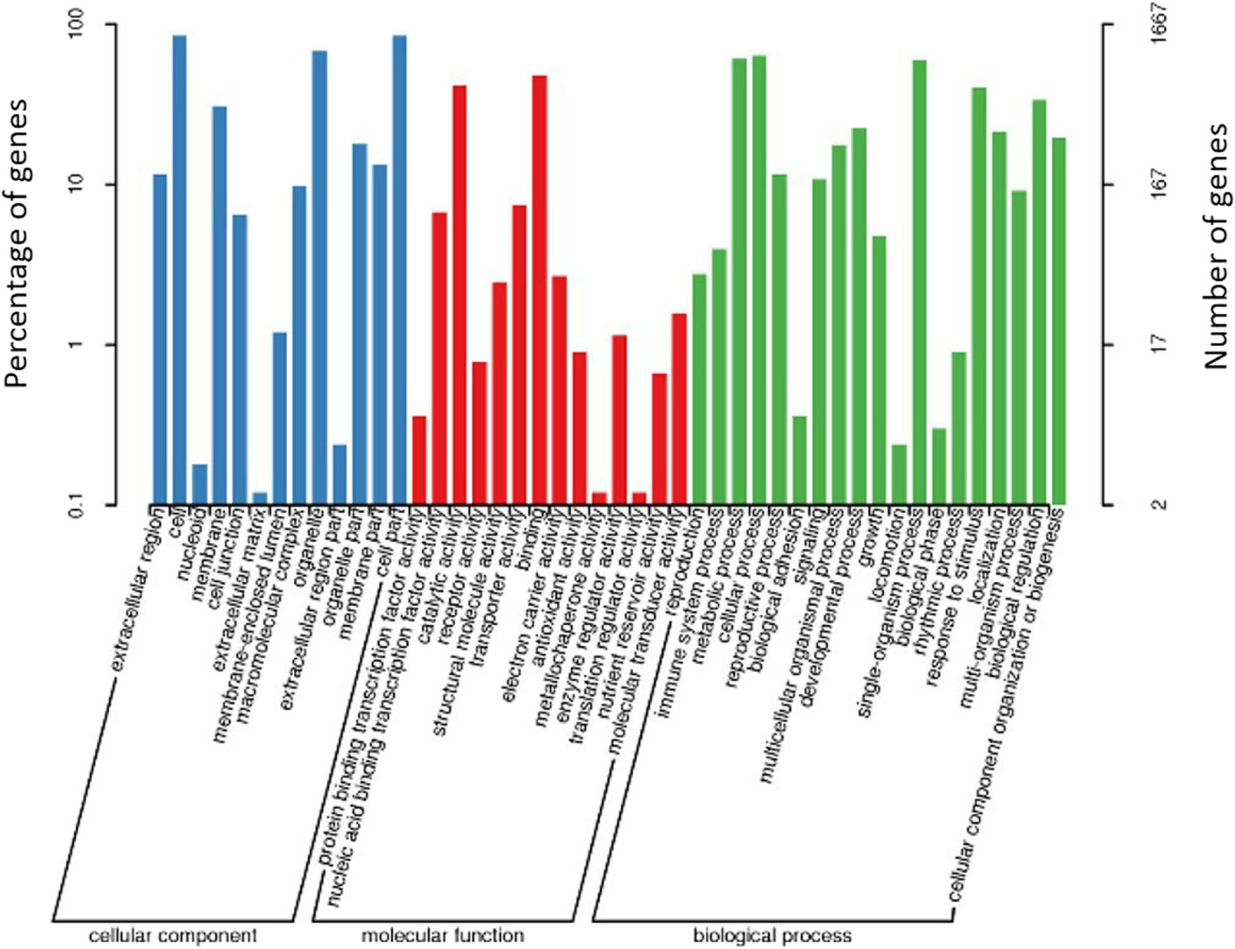
Gene ontology (GO) terms of the genes from the intersecting regions. A total of 2041 genes were divided into three categories: biological processes, cellular components, and molecular functions

To reveal the biological functions of the potential candidate genes, we performed GO and KEGG pathway enrichment analyses. Within the SNP- and InDel-associated regions, 2041 of 2044 genes could be annotated (Additional file 4: Table S3). GO annotations included 2217 terms involved in biological processes (BP, Fig. 4, Additional file 5: Table S4), 326 terms in the cellular component (CC, Fig. 4, Additional file 6: Table S5), and 799 terms in the molecular function (MF, Fig. 4, Additional file 7: Table S6). The BP terms with the highest levels of enrichment included response to nitrate (GO: 0010167), nitrate transport (GO: 0015706), negative regulation of flower development GO: 0009910), transition metal ion transport (GO: 0000041), inositol biosynthetic process (GO: 0006021), and organ development (GO: 0048513) (Additional file 5: Table S4). The CC category includes cell wall (GO: 0005618), vacuole (GO: 0005773), cytosolic small ribosomal subunit (GO: 0022627), and small ribosomal subunit (GO: 0015935) (Additional file 6: Table S5). In the MF category, the most enriched terms were sinapoyl spermidine: sinapoyl CoA N-acyltransferase activity (GO: 0080089), 2-isopropylmalate synthase activity (GO: 0003852), methyl salicylate esterase activity (GO: 0080031), nucleocytoplasmic transporter activity (GO: 0005487), and calmodulin binding (GO: 0005516) (Additional file 7: Table S6).

Moreover, 126 genes with non-synonymous mutations were identified, including 104 genes with GO annotations (Additional file 8: Table S7). Additionally, 23 genes with frame-shift mutations were identified, including 19 with GO annotations (Additional file 9: Table S8). Analysis of these annotations produced some interesting results: BnaA09g43100D (Bna.A09SFH3) and BnaC08g38270D (Bna.C08HAC12) were annotated as “flower development (GO:0009908)”; BnaC08g36330D (Bna.C08GPRI1) is related to “negative regulation of flower development (GO:0009910)”; BnaA09g44210D (Bna.A09BES1) and BnaA09g48960D are associated with “ovule development (GO:0048481)” and “carpel development (GO:0048440)”, respectively; BnaA09g42920D (Bna.A09EC1.3) was annotated as “regulation of double fertilization forming a zygote and endosperm (GO:0080155)”; and BnaA09g42700D was annotated as “specification of floral organ number (GO:0048833)”.

**Fig. 5:**
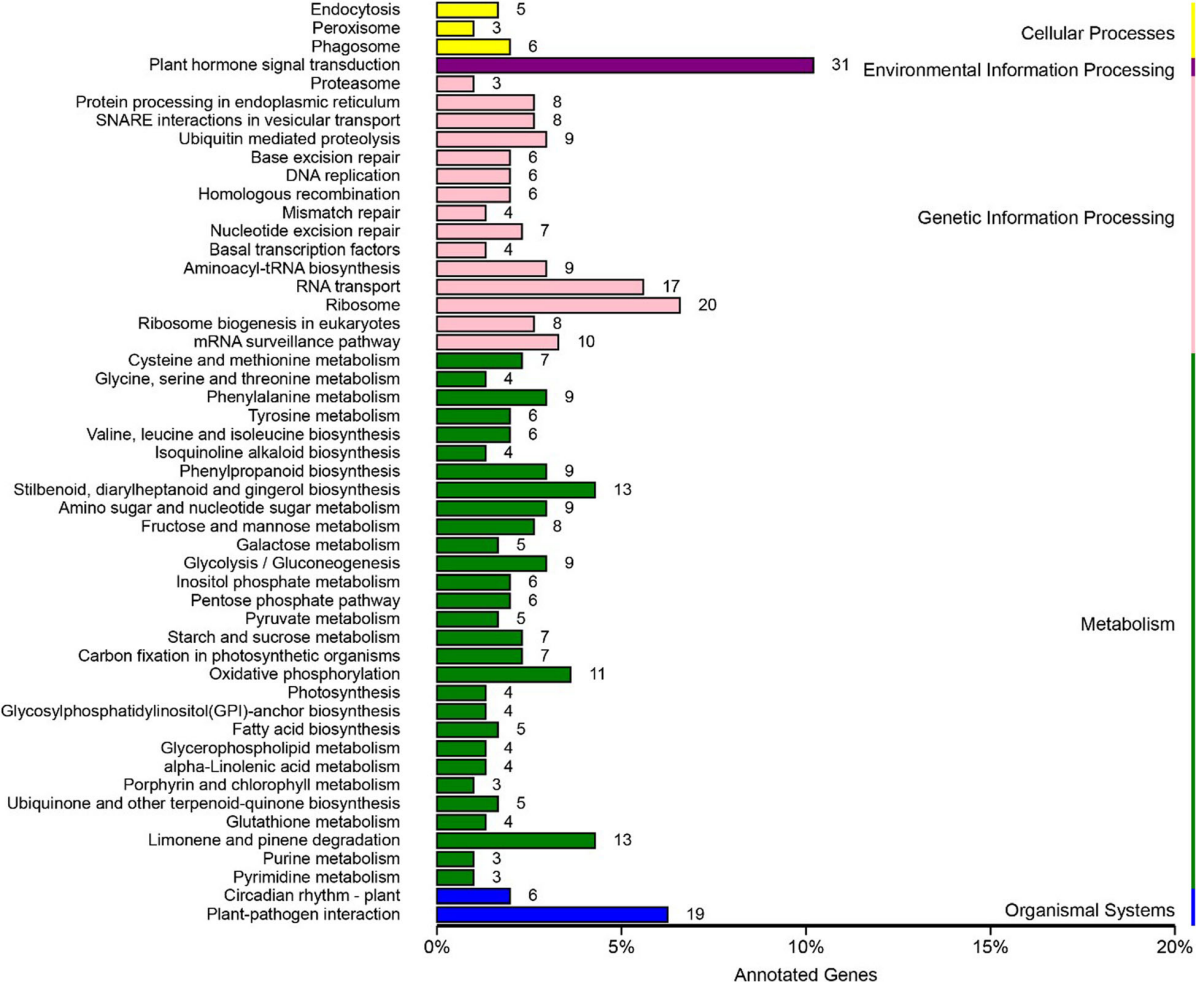
Classified KEGG pathways of the genes from the intersecting regions. The pathways were further classified into five major groups: cellular processes, environmental information processing, genetic information processing, metabolism, and organismal systems

KEGG pathway enrichment analysis revealed the four most significantly enriched pathways involving the DEGs: stilbenoid, diarylheptanoid and gingerol biosynthesis (ko00945), limonene and pinene degradation (ko00903), glycosyl phosphatidyl inositol (GPI)-anchor biosynthesis (ko00563), and isoquinoline alkaloid biosynthesis (ko00950) (Additional file 10: Table S9). These pathways were further classified into five major groups: cellular processes, environmental information processing, genetic information processing, metabolism and organismal systems (Fig. 5). Of these, the sub-group plant hormone signal transduction had the highest number of annotated genes.

### Analysis of differentially expressed genes and potential candidate gene identification

**Fig. 6:**
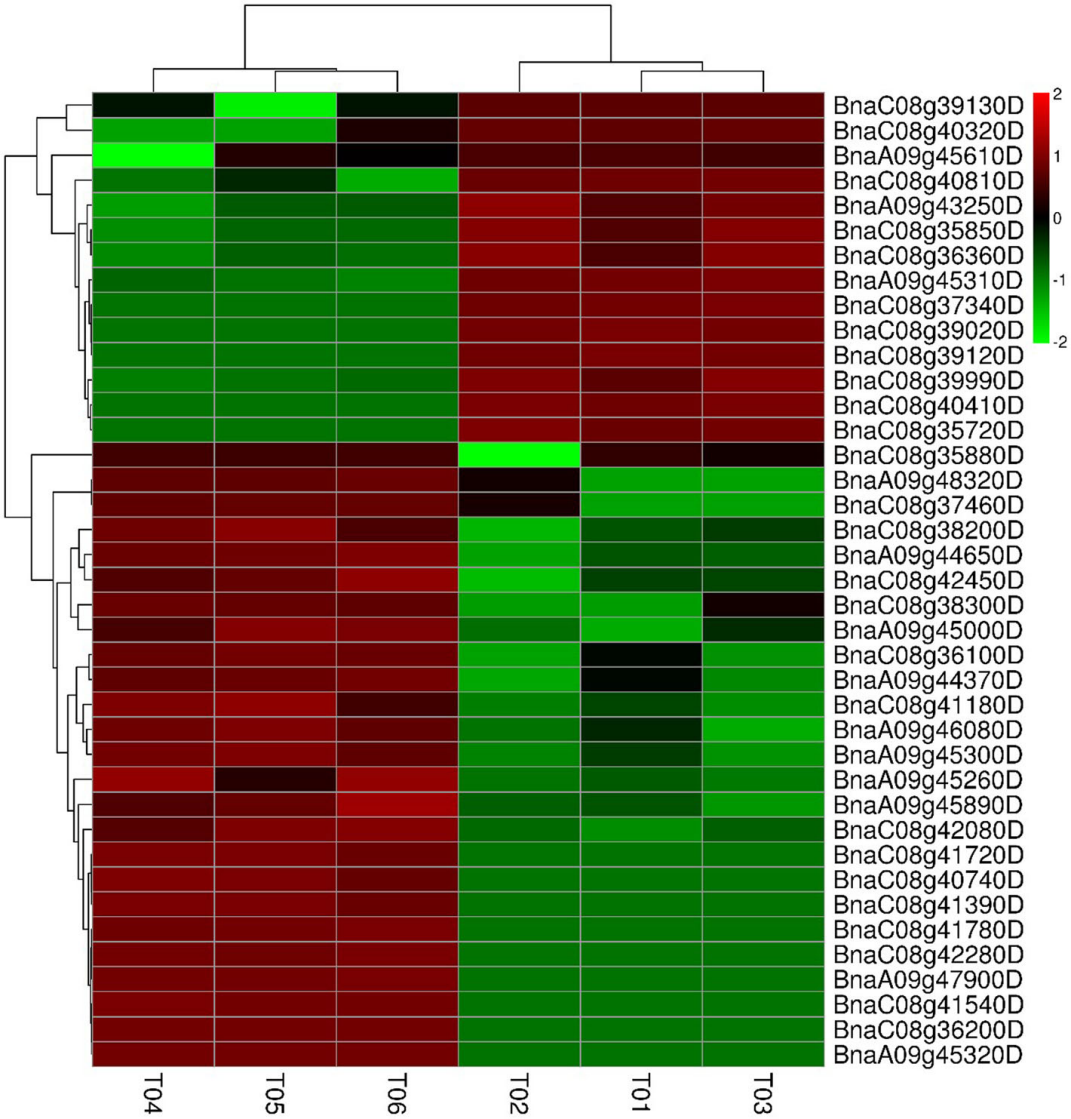
Expression patterns of 39 DEGs located in the two associated regions. The expression levels are given in log_2_(FPKM+ 1). FPKM: Fragments per Kilobase Million. T01, T02, and T03: three random samples from zws-ms at the budding stage; T04, T05, and T06: three random samples from zws-217 at the budding stage

To identify DEGs related to the multi-silique trait, we examined the expression levels of transcripts from zws-ms and compared them with those of normal plants (zms-217). A total of 129 DEGs were identified in buds, with 52.94% (67 genes) upregulated and 48.06% (62 genes) downregulated (Additional file 11: Table S10), 39 of which (Table 2) were located in the two associated regions mentioned above. Thirteen genes were located on chromosome A09, while 26 were located on chromosome C08. These 39 genes were clustered into two groups based on expression level (Fig. 6, Table 2): the first group contained 14 genes that were upregulated in multi-silique zws-ms plants, while the second group contained 25 genes that were downregulated in these plants.

**Table 2:** GO annotations of the 39 DEGs located in the two associated regions

	Gene ID	Chromosome	Expression Level	GO Annotation
1	BnaC08g40740D	C08	down	Molecular Function: translation initiation factor activity (GO:0003743); Cellular Component: cytoplasm (GO:0005737);
2	BnaA09g45890D	A09	down	Cellular Component: plasma membrane (GO:0005886); Biological Process: phosphate ion transport (GO:0006817); Cellular Component: integral component of membrane (GO:0016021); Biological Process: cellular response to phosphate starvation (GO:0016036);
3	BnaC08g42080D	C08	down	Cellular Component: plasma membrane (GO:0005886); Molecular Function: transferase activity, transferring phosphorus-containing groups (GO:0016772);
4	BnaA09g43250D	A09	up	Cellular Component: mitochondrion (GO:0005739);
5	BnaC08g36200D	C08	down	Cellular Component: chloroplast (GO:0009507); Biological Process: photorespiration (GO:0009853);
6	BnaA09g44650D	A09	down	Cellular Component: plasma membrane (GO:0005886); Biological Process: proteolysis (GO:0006508); Biological Process: lipid transport (GO:0006869); Molecular Function: peptidase activity (GO:0008233); Molecular Function: lipid binding (GO:0008289); Cellular Component: anchored component of membrane (GO:0031225);
7	BnaA09g46080D	A09	down	Cellular Component: nucleus (GO:0005634);
8	BnaC08g37340D	C08	up	Cellular Component: plasma membrane (GO:0005886); Biological Process: proteolysis (GO:0006508); Biological Process: lipid transport (GO:0006869); Molecular Function: peptidase activity (GO:0008233); Molecular Function: lipid binding (GO:0008289); Cellular Component: anchored component of membrane (GO:0031225);
9	BnaC08g41780D	C08	down	Biological Process: sulfur amino acid metabolic process (GO:0000096); Molecular Function: serine-tRNA ligase activity (GO:0004828); Molecular Function: ATP binding (GO:0005524); Cellular Component: mitochondrion (GO:0005739); Biological Process: rRNA processing (GO:0006364); Biological Process: seryl-tRNA aminoacylation (GO:0006434); Biological Process: mitochondrion organization (GO:0007005); Biological Process: cellular amino acid biosynthetic process (GO:0008652); Biological Process: serine family amino acid metabolic process (GO:0009069); Cellular Component: chloroplast (GO:0009507); Biological Process: embryo development ending in seed dormancy (GO:0009793); Biological Process: chloroplast relocation (GO:0009902); Biological Process: leaf morphogenesis (GO:0009965); Biological Process: thylakoid membrane organization (GO:0010027); Biological Process: photosystem II assembly (GO:0010207); Biological Process: vegetative to reproductive phase transition of meristem (GO:0010228); Biological Process: iron-sulfur cluster assembly (GO:0016226); Biological Process: cell differentiation (GO:0030154); Biological Process: regulation of protein dephosphorylation (GO:0035304); Biological Process: cell wall modification (GO:0042545); Biological Process: transcription from plastid promoter (GO:0042793); Biological Process: positive regulation of transcription, DNA-templated (GO:0045893); Biological Process: ovule development (GO:0048481);
10	BnaC08g40810D	C08	up	Molecular Function: protein serine/threonine kinase activity (GO:0004674); Biological Process: protein autophosphorylation (GO:0046777);
11	BnaC08g41540D	C08	down	Molecular Function: N,N-dimethylaniline monooxygenase activity (GO:0004499); Cellular Component: nucleus (GO:0005634); Biological Process: glucosinolate biosynthetic process (GO:0019761); Molecular Function: flavin adenine dinucleotide binding (GO:0050660); Molecular Function: NADP binding (GO:0050661); Biological Process: oxidation-reduction process (GO:0055114); Molecular Function: 8-methylthiopropyl glucosinolate S-oxygenase activity (GO:0080107);
12	BnaC08g40410D	C08	up	Molecular Function: Ran GTPase activator activity (GO:0005098); Cellular Component: nuclear envelope (GO:0005635); Cellular Component: vacuolar membrane (GO:0005774); Cellular Component: endoplasmic reticulum (GO:0005783); Biological Process: nucleocytoplasmic transport (GO:0006913); Biological Process: toxin catabolic process (GO:0009407); Cellular Component: chloroplast (GO:0009507); Biological Process: photomorphogenesis (GO:0009640); Biological Process: response to salt stress (GO:0009651); Biological Process: cullin deneddylation (GO:0010388); Biological Process: lateral root development (GO:0048527);
13	BnaC08g35720D	C08	up	Cellular Component: vacuolar proton-transporting V-type ATPase, V0 domain (GO:0000220); Cellular Component: mitochondrion (GO:0005739); Cellular Component: Golgi apparatus (GO:0005794); Biological Process: ATP catabolic process (GO:0006200); Cellular Component: chloroplast (GO:0009507); Molecular Function: hydrogen-translocating pyrophosphatase activity (GO:0009678); Cellular Component: plant-type vacuole membrane (GO:0009705); Molecular Function: hydrogen ion transmembrane transporter activity (GO:0015078); Biological Process: ATP synthesis coupled proton transport (GO:0015986); Biological Process: ATP hydrolysis coupled proton transport (GO:0015991); Molecular Function: ATPase activity (GO:0016887); Biological Process: cellular response to nutrient levels (GO:0031669); Biological Process: sequestering of zinc ion (GO:0032119); Biological Process: vacuolar sequestering (GO:0043181); Molecular Function: nutrient reservoir activity (GO:0045735); Biological Process: vacuolar proton-transporting V-type ATPase complex assembly (GO:0070072); Biological Process: cellular response to salt stress (GO:0071472);
14	BnaC08g42280D	C08	down	Biological Process: telomere maintenance (GO:0000723); Biological Process: double-strand break repair via homologous recombination (GO:0000724); Molecular Function: nucleic acid binding (GO:0003676); Molecular Function: ATP binding (GO:0005524); Cellular Component: nucleus (GO:0005634); Biological Process: DNA replication (GO:0006260); Cellular Component: plasmodesma (GO:0009506); Biological Process: vegetative to reproductive phase transition of meristem (GO:0010228); Molecular Function: ATP-dependent 3′-5′ DNA helicase activity (GO:0043140); Biological Process: cellular response to cold (GO:0070417); Biological Process: cellular response to abscisic acid stimulus (GO:0071215);
15	BnaC08g38300D	C08	down	Molecular Function: nucleotide binding (GO:0000166); Biological Process: mRNA splicing, via spliceosome (GO:0000398); Molecular Function: RNA binding (GO:0003723); Molecular Function: protein binding (GO:0005515); Cellular Component: nucleolus (GO:0005730); Biological Process: sugar mediated signaling pathway (GO:0010182); Cellular Component: nuclear speck (GO:0016607);
16	BnaC08g39990D	C08	up	Biological Process: MAPK cascade (GO:0000165); Molecular Function: protein serine/threonine kinase activity (GO:0004674); Molecular Function: protein serine/threonine/tyrosine kinase activity (GO:0004712); Molecular Function: ATP binding (GO:0005524); Cellular Component: nucleus (GO:0005634); Cellular Component: cytosol (GO:0005829); Biological Process: protein phosphorylation (GO:0006468); Biological Process: protein targeting to membrane (GO:0006612); Biological Process: response to cold (GO:0009409); Biological Process: response to water deprivation (GO:0009414); Biological Process: response to ethylene (GO:0009723); Biological Process: auxin-activated signaling pathway (GO:0009734); Biological Process: abscisic acid-activated signaling pathway (GO:0009738); Biological Process: brassinosteroid mediated signaling pathway (GO:0009742); Biological Process: systemic acquired resistance, salicylic acid mediated signaling pathway (GO:0009862); Biological Process: jasmonic acid mediated signaling pathway (GO:0009867); Biological Process: regulation of signal transduction (GO:0009966); Biological Process: leaf vascular tissue pattern formation (GO:0010305); Biological Process: regulation of plant-type hypersensitive response (GO:0010363); Biological Process: endoplasmic reticulum unfolded protein response (GO:0030968); Biological Process: negative regulation of defense response (GO:0031348); Biological Process: hyperosmotic salinity response (GO:0042538); Biological Process: negative regulation of programmed cell death (GO:0043069); Biological Process: defense response to fungus (GO:0050832);
17	BnaC08g36100D	C08	down	Cellular Component: cytoplasm (GO:0005737); Biological Process: ER to Golgi vesicle-mediated transport (GO:0006888);
18	BnaA09g48320D	A09	down	Molecular Function: structural constituent of ribosome (GO:0003735); Cellular Component: nucleolus (GO:0005730); Biological Process: translation (GO:0006412); Cellular Component: chloroplast (GO:0009507); Cellular Component: cytosolic large ribosomal subunit (GO:0022625);
19	BnaA09g45000D	A09	down	Biological Process: RNA splicing, via endonucleolytic cleavage and ligation (GO:0000394); Molecular Function: DNA binding (GO:0003677); Cellular Component: transcription factor TFIID complex (GO:0005669); Biological Process: DNA-templated transcription, initiation (GO:0006352); Biological Process: transcription from RNA polymerase II promoter (GO:0006366); Biological Process: cytokinin-activated signaling pathway (GO:0009736); Biological Process: jasmonic acid mediated signaling pathway (GO:0009867); Biological Process: regulation of ethylene-activated signaling pathway (GO:0010104); Molecular Function: protein heterodimerization activity (GO:0046982);
20	BnaC08g39130D	C08	up	Molecular Function: copper ion binding (GO:0005507); Molecular Function: calmodulin binding (GO:0005516); Molecular Function: ATP binding (GO:0005524); Cellular Component: mitochondrion (GO:0005739); Cellular Component: cytosol (GO:0005829); Biological Process: gluconeogenesis (GO:0006094); Biological Process: glycolytic process (GO:0006096); Biological Process: protein folding (GO:0006457); Biological Process: tryptophan catabolic process (GO:0006569); Biological Process: response to heat (GO:0009408); Biological Process: response to cold (GO:0009409); Cellular Component: chloroplast thylakoid membrane (GO:0009535); Cellular Component: chloroplast stroma (GO:0009570); Biological Process: response to high light intensity (GO:0009644); Biological Process: response to salt stress (GO:0009651); Biological Process: chloroplast organization (GO:0009658); Biological Process: indoleacetic acid biosynthetic process (GO:0009684); Cellular Component: chloroplast envelope (GO:0009941); Biological Process: isopentenyl diphosphate biosynthetic process, methylerythritol 4-phosphate pathway (GO:0019288); Biological Process: cysteine biosynthetic process (GO:0019344); Biological Process: response to endoplasmic reticulum stress (GO:0034976); Biological Process: response to hydrogen peroxide (GO:0042542); Biological Process: response to cadmium ion (GO:0046686); Cellular Component: apoplast (GO:0048046); Biological Process: ovule development (GO:0048481); Molecular Function: chaperone binding (GO:0051087); Biological Process: positive regulation of superoxide dismutase activity (GO:1901671);
21	BnaA09g45260D	A09	down	Cellular Component: chloroplast (GO:0009507);
22	BnaA09g45300D	A09	down	Molecular Function: serine-type carboxypeptidase activity (GO:0004185); Cellular Component: extracellular region (GO:0005576); Cellular Component: vacuole (GO:0005773); Biological Process: proteolysis (GO:0006508);
23	BnaC08g39020D	C08	up	Cellular Component: cytosol (GO:0005829); Cellular Component: plasmodesma (GO:0009506);
24	BnaC08g38200D	C08	down	Cellular Component: nucleus (GO:0005634); Molecular Function: oxidoreductase activity, acting on the CH-CH group of donors, NAD or NADP as acceptor (GO:0016628);
25	BnaC08g41720D	C08	down	Molecular Function: aspartic-type endopeptidase activity (GO:0004190); Cellular Component: extracellular region (GO:0005576); Cellular Component: vacuole (GO:0005773); Cellular Component: cytosol (GO:0005829); Biological Process: glycolytic process (GO:0006096); Biological Process: proteolysis (GO:0006508); Biological Process: protein targeting to vacuole (GO:0006623); Biological Process: lipid metabolic process (GO:0006629); Biological Process: water transport (GO:0006833); Biological Process: hyperosmotic response (GO:0006972); Biological Process: Golgi organization (GO:0007030); Biological Process: response to temperature stimulus (GO:0009266); Cellular Component: plasmodesma (GO:0009506); Biological Process: response to salt stress (GO:0009651); Biological Process: response to cadmium ion (GO:0046686); Biological Process: organ development (GO:0048513);
26	BnaC08g42450D	C08	down	Biological Process: response to molecule of bacterial origin (GO:0002237); Molecular Function: protein serine/threonine kinase activity (GO:0004674); Molecular Function: ATP binding (GO:0005524); Cellular Component: plasma membrane (GO:0005886); Biological Process: N-terminal protein myristoylation (GO:0006499); Biological Process: protein targeting to membrane (GO:0006612); Biological Process: membrane fusion (GO:0006944); Biological Process: response to oxidative stress (GO:0006979); Biological Process: transmembrane receptor protein tyrosine kinase signaling pathway (GO:0007169); Biological Process: systemic acquired resistance (GO:0009627); Biological Process: seed germination (GO:0009845); Biological Process: stomatal complex morphogenesis (GO:0010103); Biological Process: regulation of plant-type hypersensitive response (GO:0010363); Cellular Component: integral component of membrane (GO:0016021); Biological Process: negative regulation of programmed cell death (GO:0043069); Biological Process: protein autophosphorylation (GO:0046777); Biological Process: stamen development (GO:0048443); Cellular Component: micropyle (GO:0070825);
27	BnaC08g35850D	C08	up	Molecular Function: microtubule motor activity (GO:0003777); Molecular Function: ATP binding (GO:0005524); Cellular Component: cytoplasm (GO:0005737); Cellular Component: kinesin complex (GO:0005871); Cellular Component: microtubule (GO:0005874); Cellular Component: plasma membrane (GO:0005886); Biological Process: microtubule-based movement (GO:0007018); Molecular Function: microtubule binding (GO:0008017); Cellular Component: plasmodesma (GO:0009506);
28	BnaC08g40320D	C08	up	Molecular Function: chromatin binding (GO:0003682); Molecular Function: sequence-specific DNA binding transcription factor activity (GO:0003700); Cellular Component: nucleus (GO:0005634); Biological Process: regulation of transcription, DNA-templated (GO:0006355); Biological Process: membrane fusion (GO:0006944); Molecular Function: identical protein binding (GO:0042802); Molecular Function: sequence-specific DNA binding (GO:0043565); Biological Process: Golgi vesicle transport (GO:0048193);
29	BnaA09g45310D	A09	up	–
30	BnaC08g41180D	C08	down	Molecular Function: DNA binding (GO:0003677); Cellular Component: nucleus (GO:0005634); Molecular Function: zinc ion binding (GO:0008270);
31	BnaA09g44370D	A09	down	Molecular Function: DNA binding (GO:0003677); Molecular Function: chromatin binding (GO:0003682); Molecular Function: sequence-specific DNA binding transcription factor activity (GO:0003700); Cellular Component: nucleus (GO:0005634); Biological Process: regulation of transcription, DNA-templated (GO:0006355); Biological Process: protein targeting to membrane (GO:0006612); Biological Process: response to salt stress (GO:0009651); Biological Process: response to ethylene (GO:0009723); Biological Process: response to auxin (GO:0009733); Biological Process: response to abscisic acid (GO:0009737); Biological Process: response to gibberellin (GO:0009739); Biological Process: response to salicylic acid (GO:0009751); Biological Process: response to jasmonic acid (GO:0009753); Biological Process: positive regulation of flavonoid biosynthetic process (GO:0009963); Biological Process: regulation of plant-type hypersensitive response (GO:0010363); Biological Process: response to cadmium ion (GO:0046686);
32	BnaA09g47900D	A09	down	Molecular Function: zinc ion binding (GO:0008270);
33	BnaC08g41390D	C08	down	Cellular Component: plant-type vacuole (GO:0000325); Molecular Function: sucrose alpha-glucosidase activity (GO:0004575); Biological Process: carbohydrate metabolic process (GO:0005975); Biological Process: polyamine catabolic process (GO:0006598); Biological Process: calcium ion transport (GO:0006816); Biological Process: iron ion transport (GO:0006826); Biological Process: Golgi organization (GO:0007030); Cellular Component: plant-type cell wall (GO:0009505); Biological Process: response to wounding (GO:0009611); Biological Process: response to bacterium (GO:0009617); Biological Process: response to salt stress (GO:0009651); Biological Process: coumarin biosynthetic process (GO:0009805); Biological Process: cellular response to iron ion starvation (GO:0010106); Biological Process: response to nitrate (GO:0010167); Biological Process: nitrate transport (GO:0015706); Biological Process: brassinosteroid biosynthetic process (GO:0016132); Biological Process: cellular modified amino acid biosynthetic process (GO:0042398); Biological Process: cellular response to gibberellin stimulus (GO:0071370); Biological Process: primary root development (GO:0080022);
34	BnaA09g45610D	A09	up	Cellular Component: nucleus (GO:0005634);
35	BnaC08g35880D	C08	down	Cellular Component: cytosol (GO:0005829); Biological Process: ubiquitin-dependent protein catabolic process (GO:0006511); Cellular Component: chloroplast (GO:0009507);
36	BnaC08g36360D	C08	up	Molecular Function: nucleotide binding (GO:0000166); Molecular Function: catalytic activity (GO:0003824); Cellular Component: mitochondrial respiratory chain complex I (GO:0005747); Biological Process: ubiquitin-dependent protein catabolic process (GO:0006511); Biological Process: response to salt stress (GO:0009651); Biological Process: photorespiration (GO:0009853); Molecular Function: coenzyme binding (GO:0050662); Biological Process: response to misfolded protein (GO:0051788); Biological Process: proteasome core complex assembly (GO:0080129);
37	BnaC08g37460D	C08	down	Biological Process: mitotic cell cycle (GO:0000278); Molecular Function: RNA binding (GO:0003723); Molecular Function: polynucleotide adenylyltransferase activity (GO:0004652); Molecular Function: protein binding (GO:0005515); Cellular Component: nucleus (GO:0005634); Biological Process: transcription, DNA-templated (GO:0006351); Biological Process: RNA polyadenylation (GO:0043631);
38	BnaC08g39120D	C08	up	–
39	BnaA09g45320D	A09	down	Molecular Function: copper ion binding (GO:0005507); Molecular Function: calmodulin binding (GO:0005516); Molecular Function: ATP binding (GO:0005524); Cellular Component: mitochondrion (GO:0005739); Cellular Component: cytosol (GO:0005829); Biological Process: gluconeogenesis (GO:0006094); Biological Process: glycolytic process (GO:0006096); Biological Process: protein folding (GO:0006457); Biological Process: tryptophan catabolic process (GO:0006569); Biological Process: response to heat (GO:0009408); Biological Process: response to cold (GO:0009409); Cellular Component: chloroplast thylakoid membrane (GO:0009535); Cellular Component: chloroplast stroma (GO:0009570); Biological Process: response to high light intensity (GO:0009644); Biological Process: response to salt stress (GO:0009651); Biological Process: chloroplast organization (GO:0009658); Biological Process: indoleacetic acid biosynthetic process (GO:0009684); Cellular Component: chloroplast envelope (GO:0009941); Biological Process: isopentenyl diphosphate biosynthetic process, methylerythritol 4-phosphate pathway (GO:0019288); Biological Process: cysteine biosynthetic process (GO:0019344); Biological Process: response to endoplasmic reticulum stress (GO:0034976); Biological Process: response to hydrogen peroxide (GO:0042542); Biological Process: response to cadmium ion (GO:0046686); Cellular Component: apoplast (GO:0048046); Biological Process: ovule development (GO:0048481); Molecular Function: chaperone binding (GO:0051087); Biological Process: positive regulation of superoxide dismutase activity (GO:1901671)

Of the 39 DEGs, three (BnaA09g45320D (Bna.A09CPN10), BnaC08g39130D (Bna.C08CPN10), and BnaC08g41780D (Bna.C08SRS)) were assigned to GO categories involved in ovule development (GO: 0048481), BnaC08g42450D (Bna.C08RLK7) in stamen development (GO: 0048443) and BnaC08g41720D (Bna.C08APA1) in organ development (GO: 0048513).

### Validation of transcriptome sequencing data by qPCR

**Fig. 7:**
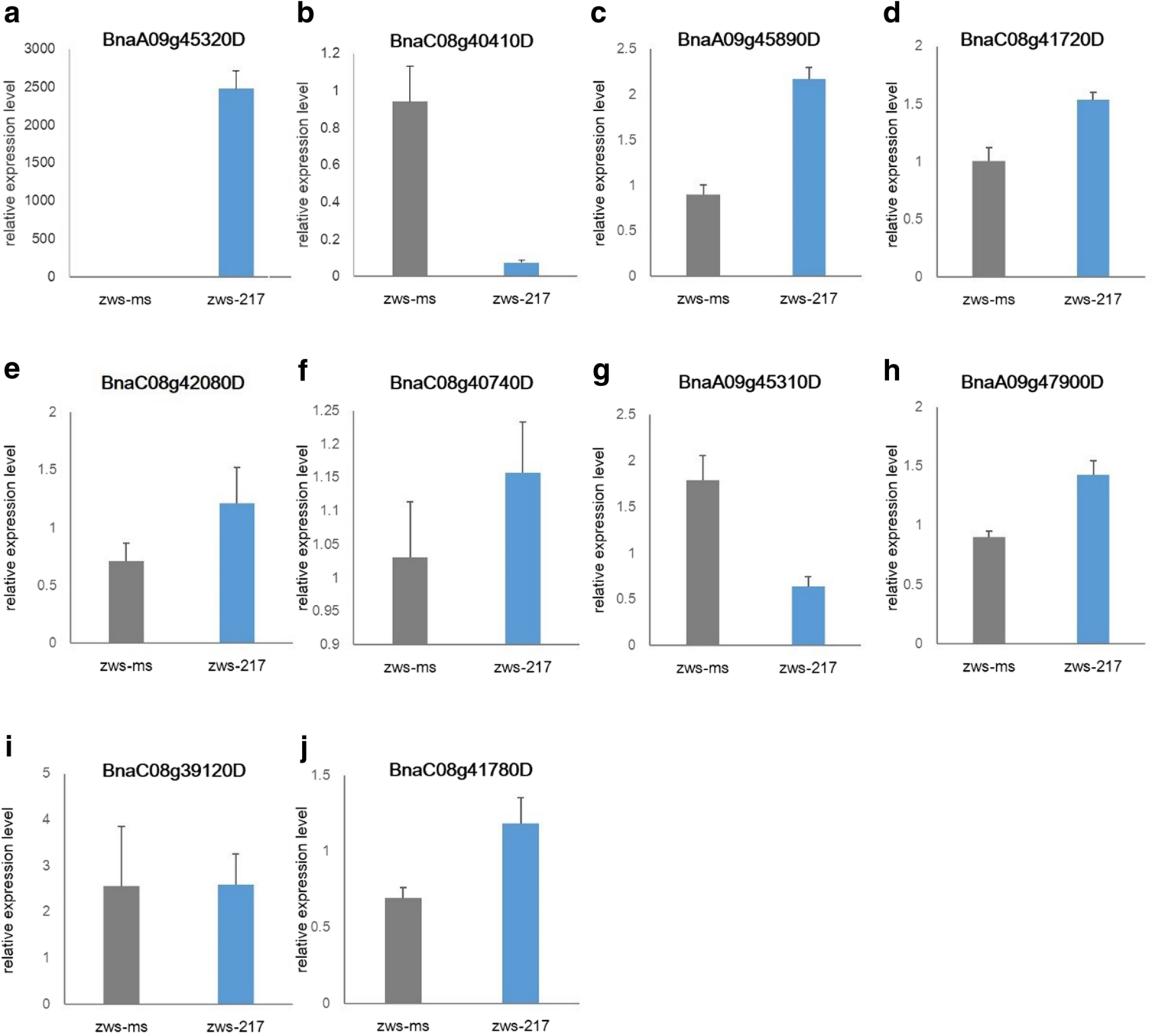
Relative expression levels (folds) of 10 selected genes determined by qPCR. Note: **a**: BnaA09g45320D; **b**: BnaC08g40410D; **c**: BnaA09g45890D; **d**: BnaC08g41720D; **e**: BnaC08g42080D; **f**: BnaC08g40740D; **g**: BnaA09g45310D; **h**: BnaA09g47900D; **i**: BnaC08g39120D; **j**: BnaC08g41780D

We validated the transcriptome sequencing data by comparing the relative transcript levels of these DEGs in zws-ms and zws-217 by qPCR. qPCR analysis of plants in buds showed that all genes except BnaC08g39120D had similar trends in expression as those observed by transcriptome sequencing, especially BnaA09g45320D and BnaC08g40410D (Figs. 6 and 7). These results further confirm the reliability of the transcriptome sequencing data described above.

### Orthologs of these 12 candidate genes in *Arabidopsis*

These 12 candidate genes mentioned above, including seven with non-synonymous mutations or frame-shift mutations, as well as the five DEGs, were then aligned with *Arabidopsis* and their orthologs (Table 3) in this well-studied model plant were identified based on The Arabidopsis Information Resource (TAIR, https://www.arabidopsis.org): (1) AT2G22370 (ortholog of BnaA09g42700D), identified as *MED18*, encodes a subunit of the mediator complex that affects flowering time and floral organ formation through *FLOWERING LOCUS C* (*FLC*) and *AGAMOUS* (*AG*); (2) AT2G21750, identified as *EGG CELL 1.3* (*EC1.3*), encodes a small cysteine-rich protein secreted by the egg cell during gamete interactions; (3) AT2G21540 is an SEC14-LIKE 3 (ATSFH3) protein; (4) AT1G19350 (*BZR2* or *BES1*) encodes brassinosteroid signalling protein that accumulates in the nucleus as dephosphorylated form in response to brassinosteroid. (5) BnaA09g45320D and BnaC08g39130D share the same ortholog, AT1G14980, known as CPN10, encoding mitochondrial-localized chaperonin 10; (6) AT1G08260, known as *TIL1* gene, encodes the catalytic subunit of DNA polymerase ε (abo4–1); (7) AT2G20570 (*GPRI1* or *GLK1*) encodes one of a pair of partially redundant nuclear transcription factors that regulate chloroplast development in a cell-autonomous manner; (8) AT1G11910 (*APA1*) encodes an aspartic proteinase that forms a heterodimer and is stable over a broad pH range; (9) AT1G11870 is identified as *OVA7* (*SRS*), of which expression products Seryl-tRNA synthetase targeted to chloroplasts and mitochondria; (10) AT1G09970 (*RLK7*) belongs to a leucine-rich repeat class of receptor-likekinase (LRR-RLKs); (11) AT1G16710 (*HAC12*) encodes an enzyme with histone acetyltransferase activity that can use both H3 and H4 histones as substrates.

**Table 3:** The 14 candidate genes and their orthologs in *Arabidopsis*

Gene in *B. napus*	orthologs in *Arabidopsis*	
	Gene ID	Description
BnaA09g42700D	AT2G22370	MEDIATOR 18 (MED18)
BnaA09g42920D	AT2G21750	EGG CELL 1.3
BnaA09g43100D	AT2G21540	SEC14-like 3, ATSFH3
BnaA09g44210D	AT1G19350	BRASSINAZOLE-RESISTANT 2 (BZR2), BRI1-EMS-SUPPRESSOR 1 (BES1)
BnaA09g45320D	AT1G14980	chaperonin 10 (CPN10)
BnaA09g48960D	AT1G08260	TILTED 1 (TIL1)
BnaC08g36330D	AT2G20570	GBF’s pro-rich region-interacting factor 1 (GPRI1), GOLDEN2-LIKE 1 (GLK1)
BnaC08g38270D	AT1G16710	histone acetyltransferase of the CBP family 12 (HAC12)
BnaC08g39130D	AT1G14980	chaperonin 10 (CPN10)
BnaC08g41720D	AT1G11910	aspartic proteinase A1 (APA1)
BnaC08g41780D	AT1G11870	Seryl-tRNA synthetase (SRS), OVULE ABORTION 7 (OVA7)
BnaC08g42450D	AT1G09970	LRR XI-23, RECEPTOR-LIKE KINASE 7 (RLK7)

## Discussion

In the current study, we constructed NILs to establish the two extreme DNA pools (multi-silique pool and single-silique pool), instead of the frequently used F_2_ [28–30], doubled haploid (DH) [25, 31], or recombinant inbred line (RIL) [32, 33] populations. BSA [20] combined with NGS [34], which has been successfully used in various crops [29, 30, 32, 35, 36]. Here, the WGR produced abundant, high-quality data. After filtration, 1,135,690 HQ SNPs and 289,832 InDels were identified and used to conduct association analysis. These markers were clearly superior to traditional markers in terms of number, efficiency, and quality: as we expected, these markers were much denser than SSR or other markers and the combination of BSA and WGR saved the labors and shortened the whole process. As we were unable to use the SNP-index method in this study, we used the ED algorithm instead, followed by the identification of three SNP-associated regions, including one on chromosome A09 and two on chromosome C08. The two regions on chromosome C08 were very close to each other (only 0.14 Mb apart), and the smaller region was only 0.07 Mb long, suggesting that it is likely a minor site. Moreover, we identified two InDel-associated regions. Taken together, the SNP-associated region data were highly consistent in terms of position with those of the InDel-associated regions, confirming the reliability of both data sets. Therefore, the two intersecting regions are considered to be highly associated with the multi-silique trait, representing the only difference between the zws-ms and zws-217 pools. Preliminary analysis indicated that these two regions contain 2044 genes, including 126 with non-synonymous mutations and 23 with frame-shift mutations.

Since these genes were identified by WGR, they had small differences in sequence, which consequently led to changes in the amino acid sequences, structures, and final functions of the proteins. Since zws-ms and zws-217 are NILs that only differ from each other in the multi−/single-silique trait, we reasoned that these genes are associated with this trait and were therefore selected as candidate genes. However, some genes might have differed between the two pools in terms expression levels rather than nucleotide sequence. Therefore, we performed transcriptome sequencing from buds in plants to uncover the DEGs between lines, which we then confirmed by qPCR. We identified 129 DEGs between zws-ms and zws-217, which are distributed on all chromosomes except chromosome A06. Among the 129 DEGs, 39 are located in the two important trait-associated regions identified in this study and are therefore considered highly related to the multi-silique trait. These DEGs were classified into two groups based on expression level, including one containing upregulated genes and the other containing downregulated genes.

A comprehensive understanding of the functions of the 2044 genes in the two intersecting regions is crucial for candidate gene selection. Unlike standard agronomic traits (such as plant height and flowering time), which are relatively well-studied and for which some candidate genes are available for reference, the multi-silique trait analyzed in this study has not been studied on genomic or molecular level in detail to date. Thus, GO annotation and KEGG pathway analysis provided important clues for candidate gene identification. Among the 2044 genes within the two associated regions, 2041 were successfully annotated. KEGG pathway analysis provided a general view of the genes in these regions. Most enriched KEGG pathways were involved in the biosynthesis or degradation of some secondary metabolites.

As we were unable to relate this information to the multi-silique trait using established knowledge, we conducted a GO analysis. Eight of these DEGs were annotated in the category “negative regulation of flower development (GO: 0009910)” and 221 genes were involved in “organ development (GO: 0048513)”. This information is too general to reveal an association with flower/silique development. In addition, most genes within the two regions were the same between the two DNA pools. Thus, more attention should be paid to genes showing polymorphism between zws-ms and zws-217 plants, specifically genes harboring non-synonymous mutations or frame-shift mutations. Furthermore, 104 of the 126 genes with non-synonymous mutations and 19 of the 23 genes with frame-shift mutations were represented in the GO database. We examined each of their annotations and identified seven interesting genes, which were annotated to ovule, carpel or stamen development. The NILs zws-ms and zws-217 differ only in terms of flower and silique morphology and structure. Therefore, genes that differ in zws-ms versus zws-217 at the genomic level are thought to encode diverse proteins, suggesting they are excellent candidate genes for the multi-silique trait.

At the transcriptome level, we focused on DEGs, especially those located in the associated regions mentioned above. Thirty-nine out of 129 DEGs were found in the two intersecting regions, among which five genes were annotated to the ovule or stamen development. This annotation information strongly suggests that these DEGs are involved in the multi-silique trait, as zws-ms flowers have obviously abnormal morphology, consequently leading to the formation of abnormal fruits.

Then, we aligned these selected genes to the *Arabidopsis*, screening for homologous genes sharing the highest sequence identity with them, but did not identify more potential candidate genes other than these 12 genes already mentioned (BnaA09g43100D, BnaC08g38270D, BnaC08g36330D, BnaA09g44210D, BnaA09g48960D, BnaA09g42920D and BnaA09g42700D with non-synonymous mutations or frame-shift mutations, as well as BnaA09g45320D, BnaC08g39130D, BnaC08g41780D, BnaC08g42450D and BnaC08g41720D with different expression levels). Based on analysis of these orthologs in *Arabidopsis* and their roles in plants, the annotated information of these candidate genes are confirmed: (1) BnaA09g42700D is found much shorter than its *Arabidopsis* ortholog AT2G22370 (*MED18*) in length, but in their overlapping region, they share 100% identity of nucleotide sequence. Mutation of *MED18* in *Arabidopsis* significantly delayed its flowering time [37]. *FLC* encodes an MADS-box protein and is a repressor for flowering [38]; *AG* is a floral meristem identity gene, which specifies carpel and stamen identity in the flower [39, 40]. Thus, BnaA09g42700D could probably act on flowering indirectly. (2) Disruption of AT1G11870 results in ovule abortion in *Arabidopsis* [41]. So the different expression of BnaC08g41780D between zws-ms and zws-217 may result some unknown process in fruit development. (3) The knockdown of *HAC1* causes disturbances in flower morphology in the *Arabidopsis*, such as a modified petal shape or even absence of petals [42]. *HAC12* has high identity with *HAC1*, but what morphology changes it can make are still unknown. (4) *SEC14-LIKE 3* (*ATSFH3*) is predominantly expressed in the flowers and involved in the transfer of phosphatidylinositol or phosphatidylcholine phospholipids during flower development in *Arabidopsis* [43, 44]. In chickpea (*Cicer arietinum* L.), *SEC14-LIKE3* is also considered a potential candidate flowering time-regulating genes by mapping [45]. (5) *EC1.3* triggers sperm cell activation during double fertilization and knockdown of it reduces seed number in siliques [46]. (6) The brassinosteroid signal is found involved in ovule initiation and development, by regulating the expression level of *BZR1*, of which dephosphorylation increases ovule and seed number in *Arabidopsis* [47]. In this study, BnaA09g44210D has non-synonymous mutation between zws-ms and zws-217, probably causes multi-silique trait by some undiscovered pathways. (7) *ATGLK1* (*GPRI1*) can regulate the silique number in *Arabidopsis*: the *glk1/glk2* double mutant realized only about 1/3 of the mean silique number per plant found in the wild type, due to changes in silique wall and leaf photosynthesis [48]. (8) The *Arabidopsis* mutant *til1–4/til1–4* has altered floral phyllotaxis, reduced ovule number, abnormally developed ovules, and reduced fertility [49]. (9) *APA1*: In *Cynara cardunculus*, the aspartic proteinase cardosin B is found playing an important role in ovule function [50]. (10) *RLK7*: Stamen development depends on a teamwork of receptor-like kinases [51, 52]. (11) CPN10 is involved in response to many physiological process, such as seed abortion in plants [53]. Although no report directly leads to multi-silique trait so far, these orthologs are all related to flower development, flower morphology change, fruit development and even the number of ovules/seeds. It is due to the scarcity of the multi-silique trait to some extent. Moreover, this multi-silique trait is theoretically more complex, since it is controlled by multi-loci. Anyhow, these clues validated the 12 candidate genes.

In summary, the unique multi-silique rapeseed zws-ms, examined in this study represents an enriched rapeseed germplasm resource, which is considered the basis of crop breeding. Thus far, 12 candidate genes in two genomic regions associated with the multi-silique trait have been identified, including seven genes with sequence changes and five genes with expression level differences between the multi-silique and single-silique NILs. This information lays the foundation for future research, such as candidate genes isolation, their functional verification and so on. Several unanswered questions remain. A previous study suggested that the multi-silique is likely controlled by three allelic genes [19], but in the current study, we only identified two associated regions, which harbor no more than two independent genes. This finding suggests that one more gene/factor remains unidentified. We also noted that the multi-silique trait is highly sensitive to the environment. For example, when zws-ms plants were planted in Xindu (altitude of 472 m, 30°47′10″N 104°12′12″E), the multi-silique trait was continuously stable for years; but when they were grown in Ma’erkang (altitude of 2540 m, 31°94′15″N 102°14′29″E), the multi-silique trait disappeared, and all plants had normal siliques (Additional file 12: Fig. S2). Interestingly, once we transferred zws-ms seeds harvested in Ma’erkang back to Xindu, the multi-silique trait was recovered. By contrast, zws-217 maintained the single-silique phenotype at both locations.

Thus, there might be some epigenetic reason for this trait, such as methylation of a base that does not alter the DNA sequence of this underlying locus but is strongly affected by environmental factors. We noticed that there were several genes in the environmental response category in the associated regions with SNP/InDel variations between the two DNA pools: BnaA09g45600D was annotated as “cellular response to water deprivation (GO: 0042631)” and BnaA09g44900D as “response to cold (GO: 0009409)”. Moreover, several DEGs were also annotated to environment response, like BnaA09g45320D was also annotated as “response to cold” and so on. Since these genes are responsive to environmental factors, such as cold, water, and light intensity, their functions vary when their sequences or expression levels change. Thus, they may have different effects under different environmental conditions. Furthermore, Xindu is located in the Sichuan Basin, which has a humid subtropical monsoon climate (with an annual average temperature of 16.2 °C), whereas Ma’erkang is located in a mountainous area in western Sichuan, where the annual average temperature is only 8.6 °C. The environmental factor with the greatest difference between the two locations is temperature. Thus, we propose that temperature is the most important environmental factor affecting the multi-silique trait in zws-ms, although other factors such as light intensity are also likely involved in regulating this trait.

## Conclusions

A novel trait in rapeseed, multi-siliques, was investigated at the genome level by association analysis based on WGR, followed by analysis at the transcriptome level. The two regions associated with this trait contain seven genes with non-synonymous mutations or frame-shift mutations annotated to floral organ-related GO terms, as well as five other vital DEGs. These genes are interesting candidate genes for the multi-silique trait.

## Methods

### Plant materials and population development

The original multi-silique rapeseed line was discovered at the Crop Research Institute, Sichuan Academy of Agricultural Sciences from a population derived from a cross between *B. napus* and *B. rapa*. Multi-silique plants were grown and self-pollinated for six successive generations to obtain the homozygous multi-silique zws-ms plants. Single-silique offspring were continuously backcrossed for six generations with zws-ms as the recurrent parent, followed by six continuous generations of self-pollination to obtain the near-isogenic line zws-217 with the single-silique phenotype (Fig. 1). The NILs zws-217 and zws-ms were grown in the field under standard conditions at the Sichuan Academy of Agricultural Sciences in the Xindu District of Sichuan Province, China.

### DNA preparation and pool construction

The rate of multi-silique formation per zws-ms plant was determined by phenotyping at the full-bloom stage (BBCH 67). The multi-silique rate per plant was calculated as:

$$ \mathrm{multi}-\mathrm{silique}\ \mathrm{rate}=\frac{\mathrm{number}\ \mathrm{of}\ \mathrm{three}-\mathrm{pistiled}\ \mathrm{flowers}\ \mathrm{per}\ \mathrm{plant}}{\mathrm{total}\ \mathrm{number}\ \mathrm{of}\ \mathrm{flowers}\ \mathrm{in}\ \mathrm{this}\ \mathrm{plant}} $$

Thirty plants with high multi-silique rates were selected for DNA isolation, while 30 zws-217 plants were randomly selected. Fresh leaves were sampled from these plants (zws-ms and zws-217) and subjected to DNA extraction by the cetyltrimethyl ammonium bromide (CTAB) method [54]. RNA contamination was removed from each sample using RNase A. The DNA was quantified using a NanoDrop 2000 Spectrophotometer (Thermo Scientific, USA). Samples with optical density 260/280 ratios ranging from 1.8 to 2.2 were considered adequate. Equal quantities of DNA were pooled by line (multi-silique and single-silique), with 30 samples per line, to a final concentration of 40 ng/μl.

### Whole genome re-sequencing

Pooled DNA samples were sheared into ~ 350 bp fragments using a Covaris S2/E210 DNA Shearing kit. The sheared DNA was end-repaired and a single nucleotide (A) overhang was subsequently added to the repaired fragments by adding a Klenow DNA polymerase Fragment (3′ → 5′ exo–) (New England Biolabs, Ipswich, MA, USA) and dATP at 37 °C. Barcodes and Illumina sequencing adapters were ligated to the A-tailed fragments using T4 DNA Ligase (Takara, Dalian, China). PCR was performed on both pooled samples using diluted prepared (sheared and ligated) DNA, deoxyribonucleotide triphosphate, Q5® High-Fidelity DNA Polymerase, and PCR primers. The PCR products were purified using Agencourt AMPure XP Beads (Beckman Coulter, High Wycombe, UK). Fragments 300 to 500 bp in length (including barcodes and adaptors) were excised and purified using a QIAquick Gel Extraction Kit (Qiagen, Hilden, Germany). Gel-purified products were diluted for pair-end sequencing (150 bp on each end) on an Illumina HiSeq system following the standard protocol (Illumina, Inc.; San Diego, CA, USA) at the Biomarker Technologies Corporation (Beijing, China). The sequencing depth was roughly 30 × .

### SNP calling

Low-quality reads with quality scores <20e were filtered out and raw reads were sorted according to their barcode sequences. After the barcodes were trimmed, clean HQ reads were mapped to the Brassica_napus.v4.1.fa genome (http://www.genoscope.cns.fr/brassicanapus/data/) using the Burrows-Wheeler Aligner software [55]. SAMtools [56] was used to mark duplicates, and GATK [57] was used for local realignment and base recalibration. An SNP set was formed by combining the results of analysis with GATK and SAMtools via SNP calling using default parameters. SNPs identified between the two lines were regarded as polymorphic for association analysis.

### Association analysis

Euclidean distance (ED) association analysis is a method that calculates ED values (quadratic sum root of differences between bulks from the depth of four types of bases) and is satisfied by ED. In theory, the higher the ED value is, the closer the object site [25]. The ED values were calculated as follows [27]:

$$ \mathrm{ED}=\sqrt{{\left({\mathrm{A}}_{\mathrm{ms}}-{\mathrm{A}}_{217}\right)}^2+{\left({\mathrm{C}}_{\mathrm{ms}}-{\mathrm{C}}_{217}\right)}^2+{\left({\mathrm{G}}_{\mathrm{ms}}-{\mathrm{G}}_{217}\right)}^2+{\left({\mathrm{T}}_{\mathrm{ms}}-{\mathrm{T}}_{217}\right)}^2} $$

In this formula, A_ms_, C_ms_, G_ms_, and T_ms_ represent the depth of bases A, C, T, and G on a site in the multi-silique pool, and A_217_, C_217_, G_217_, and T_217_ represent the depth of bases A, C, T, and G on a site in the single-silique pool, respectively. To remove background noise, the original ED^5^ value was used, and the adjusted values were fit using the DISTANCE method. Regions over a threshold value were considered to be trait-related candidate regions. The associated threshold value was determined based on ED + 3SD (standard deviation) [27]. InDel-associated regions were obtained via a similar method.

### RNA isolation for transcriptome analysis

Three individual plants of the zws-217 (T04, T05, and T06) line were randomly selected for RNA isolation. Eight to ten buds (BBCH 57) per plant were randomly selected and sampled. For the zws-ms, three plants (T01, T02, and T03) were selected, and buds were sampled from each plant. All buds were quick-frozen and stored in liquid nitrogen. Before RNA isolation, the buds from three zws-ms plants were sliced with tweezers and observed. Only buds containing three pistils were considered to be correct multi-silique buds and used for RNA isolation. Buds from each plant were mixed to isolate the RNA, yielding three samples from zws-217 (T04, T05, and T06) and three samples from zws-ms (T01, T02, and T03). Since zws-217 and zws-ms were NILs, and these plants differ from each other only in the multi-silique trait, we reasoned that DEGs between these lines might be related to this trait. Total RNA was isolated using an RNA Isolation Kit (Tiangen, Beijing, China) and the concentration measured using a NanoDrop 2000 (Thermo). RNA integrity was assessed using an RNA Nano 6000 Assay Kit and the Agilent Bioanalyzer 2100 system (Agilent Technologies, CA, USA).

### RNA library preparation and transcriptome sequencing

For each tissue sample, 1 μg RNA was used as input material for RNA sample preparation. Sequencing libraries were generated using a NEBNext Ultra™ RNA Library Prep Kit for Illumina (NEB, USA) following the manufacturer’s instructions with index codes added to associate sequences to each sample. Briefly, mRNA was purified from total RNA using poly-T oligo-attached magnetic beads. Fragmentation was carried out using divalent cations under elevated temperature in NEBNext First Strand Synthesis Reaction Buffer (5×). First-strand cDNA was synthesized using random hexamer primer and M-MuLV Reverse Transcriptase. Second-strand cDNA synthesis was subsequently performed using DNA Polymerase I and RNase H. The remaining overhangs were converted into blunt ends using exonuclease/polymerase. After adenylation of the 3′ ends of DNA fragments, a NEBNext Adaptor with a hairpin loop structure was ligated to prepare for hybridization. To select 240 bp cDNA fragments, the library fragments were purified using the AMPure XP system (Beckman Coulter, Danvers, MA USA). The size-selected, adaptor-ligated cDNA was incubated with 3 μl USER Enzyme (NEB, USA) at 37 °C for 15 min, followed by 5 min at 95 °C. PCR was then performed with Phusion High-Fidelity DNA polymerase, universal PCR primers, and an Index (X) Primer. The PCR products were purified (AMPure XP system) and library quality assessed on the Agilent Bioanalyzer 2100 system.

Clustering of index-coded samples was performed on a cBot Cluster Generation System using a TruSeq PE Cluster Kit v4-cBot-HS (Illumina) according to the manufacturer’s instructions. After cluster generation, the libraries were sequenced on the Illumina HiSeq X-ten platform and paired-end reads were generated.

Raw reads in fastq format were initially processed using in-house Perl scripts. During this step, clean reads were obtained by removing adapter sequences, reads containing poly-N, and low-quality reads. At the same time, the Q20, Q30, GC-content, and sequence duplication levels of the clean reads were calculated. All downstream analyses were based on clean HQ reads.

Clean reads were mapped to the reference genome sequence, and only reads with a perfect match or one mismatch were further analyzed and annotated based on the reference genome (http://www.genoscope.cns.fr/brassicanapus/data/). Tophat2 tools (parameters: --read-mismatches 2 --read-edit-dist 2 --max-intron-length 5,000,000 --library-type fr-unstranded --mate-inner-dist 40) were used to map clean reads to the reference genome.

### Differential expression analysis

Differential expression analysis of the two lines was performed using the DESeq R package [58]. DESeq provides statistical models based on the negative binomial distribution to identify DEGs using gene expression data. The resulting *P* values were adjusted using the Benjamini and Hochberg’s approach for controlling the false discovery rate. Genes with an adjusted fold change of 4 and a *p*-value ≤0.01 were considered differentially expressed.

### Reverse-transcription quantitative PCR (qPCR) validation

To confirm the validity of the DEGs identified by transcriptome sequencing, qPCR was performed. Reverse transcription to cDNA was conducted using a PrimeScript First-strand cDNA Synthesis Kit (Takara, Dalian, China). The relative transcript levels of 10 selected DEGs (at the budding stage BBCH 57) were re-examined by qPCR, with *BnACTIN* used as the internal control. The primer sequences (Additional file 13: Table S11) for the genes were designed with Premier Primer 3.0 software. All qPCR was performed on a Roche480 instrument (Roche, Basel, Switzerland). Amplification reactions were performed as follows: an initial denaturation step at 95 °C for 3 min, 39 cycles at 95 °C for 10 s, 58 °C for 30 s, and 72 °C for 30 s, a final extension at 72 °C for 10 min and hold at 15 °C.

## Additional files

Additional file 1: **Figure S1.** Whole genome with associated regions. (DOCX 209 kb)

Additional file 2: **Table S1.** Summary of the transcriptome sequencing data. (DOCX 15 kb)

Additional file 3: **Table S2.** Information about the mapped reads based on the transcriptome sequencing data. (DOCX 15 kb)

Additional file 4: **Table S3.** Annotations of the 2041 genes in the two intersection associated regions. (XLSX 340 kb)

Additional file 5: **Table S4.** GO enrichment in biological processes (BP) category of the genes. (XLSX 123 kb)

Additional file 6: **Table S5.** GO enrichment in cellular components (CC) category of the genes. (XLSX 26 kb)

Additional file 7: **Table S6.** GO enrichment in molecular function (MF) category of the genes. (XLSX 50 kb)

Additional file 8: **Table S7.** GO annotations of the 104 genes with non-synonymous mutation SNPs in the associated regions. (DOCX 34 kb)

Additional file 9: **Table S8.** GO annotations of the 19 genes with frame-shift mutation InDels in the associated regions. (DOCX 17 kb)

Additional file 10: **Table S9.** KEGG enrichment of the 2041 genes in the intersection associated regions. (XLSX 12 kb)

Additional file 11: **Table S10.** Information about the DEGs between zws-ms and zws-217. (DOCX 40 kb)

Additional file 12: **Figure S2.** Stability of the multi-silique trait under one environment and variation of this trait across different environments. (DOCX 1840 kb)

Additional file 13: **Table S11.** Primers used for qPCR. (DOCX 15 kb)

## Abbreviations

BSA: Bulked-segregant analysis
CTAB: Cetyltrimethyl ammonium bromide
DEG: Differentially expressed gene
DH: Doubled haploid
ED: Euclidean Distance
GO: Gene ontology
HQ: High quality
InDel: Insertion/deletion
KEGG: Kyoto Encyclopedia of Genes and Genomes
NGS: Next-generation sequencing
NIL: Near-isogenic line
qPCR: Reverse-transcription quantitative PCR
QTL: Quantitative trait locus/loci
RAPD: Random amplified polymorphic DNA
RIL: Recombinant inbred line
SLAF-seq: Specific length amplified fragment sequencing
SNP: Single nucleotide polymorphism
SRAP: Sequence related amplified polymorphism
SSR: Simple sequence repeat; WGR: Whole genome re-sequencing
