# Supplementary figures and images for: Identification of genomic regions associated with multi-silique trait in Brassica napus
Source: BMC Genomics. 2019 Apr 23;20:304. doi: 10.1186/s12864-019-5675-4 (PMC6480887; doi:10.1186/s12864-019-5675-4)

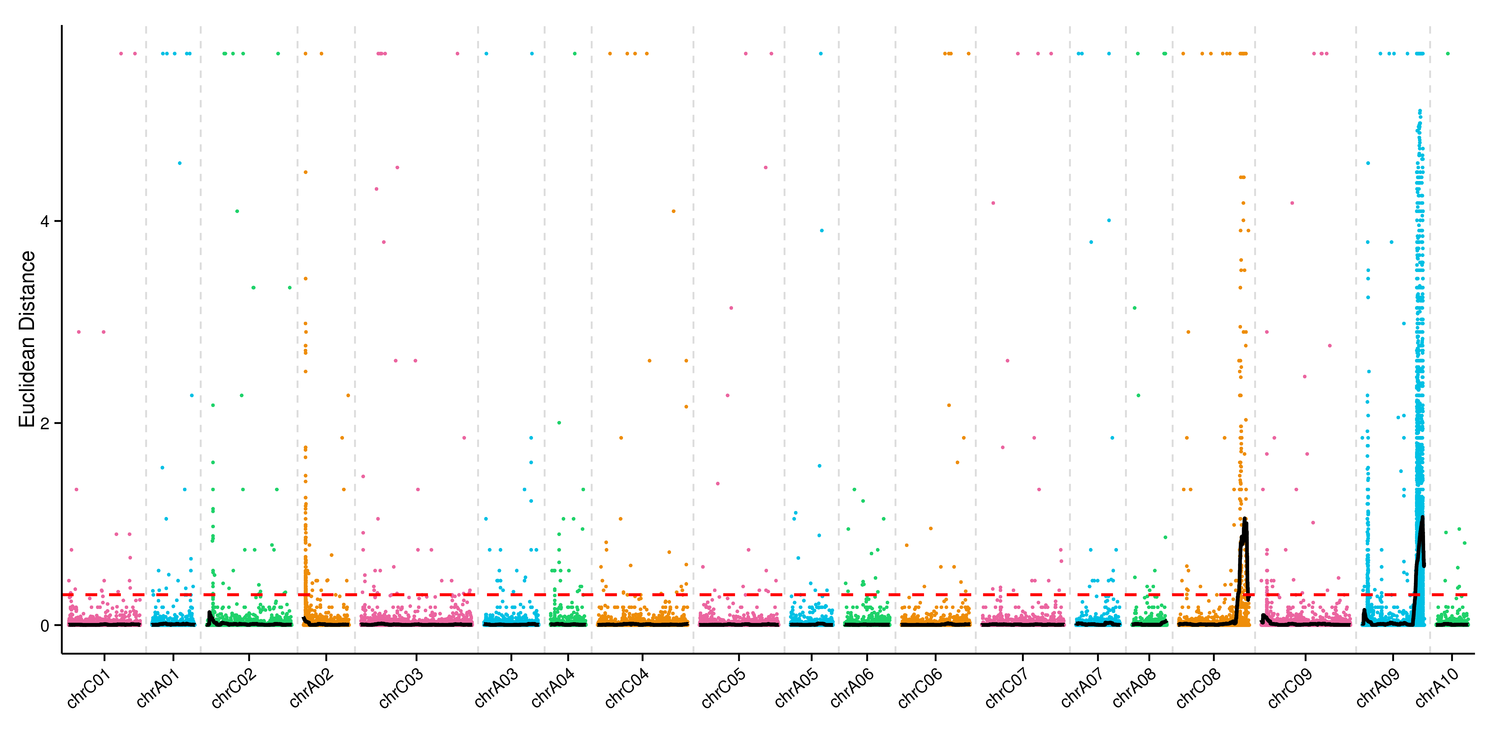


Additional file1: Fig S1. The whole genome with two identified associated regions.

Supplement: Supplementary file 1 — Figure S1. Whole genome with associated regions. (DOCX 209 kb) [file 12864_2019_5675_MOESM1_ESM.docx]
