# Supplementary material for: Identification of genomic regions associated with multi-silique trait in Brassica napus
Source: BMC Genomics. 2019 Apr 23;20:304. doi: 10.1186/s12864-019-5675-4 (PMC6480887; doi:10.1186/s12864-019-5675-4)
Supplement: Supplementary file 2 — Table S1. Summary of the transcriptome sequencing data. (DOCX 15 kb) [file 12864_2019_5675_MOESM2_ESM.docx]

Additional file 2: Table S1. Summary of the transcriptome sequencing data

| **Sample** | **Clean reads** | **Clean bases** | **GC Content** | **%≥Q30** |
| --- | --- | --- | --- | --- |
| T01 | 27,988,798 | 8,342,636,670 | 46.67% | 89.85% |
| T02 | 33,972,932 | 10,138,074,420 | 47.72% | 91.58% |
| T03 | 41,826,443 | 12,487,251,074 | 47.51% | 91.62% |
| T04 | 39,062,063 | 11,657,288,984 | 47.18% | 89.73% |
| T05 | 41,486,437 | 12,383,790,994 | 47.21% | 90.36% |
| T06 | 35,568,484 | 10,595,114,562 | 47.08% | 90.12% |

Note: T1, T2, and T3: bud samples from three independent plants in the zws-ms population at the budding stage; T4, T5, and T6: bud samples from three independent plants in the zws-217 population at the budding stage
