# Supplementary material for: Identification of genomic regions associated with multi-silique trait in Brassica napus
Source: BMC Genomics. 2019 Apr 23;20:304. doi: 10.1186/s12864-019-5675-4 (PMC6480887; doi:10.1186/s12864-019-5675-4)
Supplement: Supplementary file 3 — Table S2. Information about the mapped reads based on the transcriptome sequencing data. (DOCX 15 kb) [file 12864_2019_5675_MOESM3_ESM.docx]

Additional file 3: Table S2. Information about the mapped reads based on the transcriptome sequencing data.

| **Sample** | **Total Reads** | **Mapped Reads** | **Unique Mapped Reads** | **Multiple Mapped Reads** | **Reads Mapped to '+'** | **Reads Mapped to '-'** |
| --- | --- | --- | --- | --- | --- | --- |
| T01 | 55,977,596 | 40,616,347 (72.56%) | 33,516,649 (59.88%) | 7,099,698 (12.68%) | 17,896,621 (31.97%) | 17,931,699 (32.03%) |
| T02 | 67,945,864 | 51,659,641 (76.03%) | 43,096,179 (63.43%) | 8,563,462 (12.60%) | 22,909,005 (33.72%) | 22,930,137 (33.75%) |
| T03 | 83,652,886 | 63,288,085 (75.66%) | 53,361,914 (63.79%) | 9,926,171 (11.87%) | 28,271,478 (33.80%) | 28,305,083 (33.84%) |
| T04 | 78,124,126 | 56,100,720 (71.81%) | 46,941,345 (60.09%) | 9,159,375 (11.72%) | 24,950,376 (31.94%) | 24,976,493 (31.97%) |
| T05 | 82,972,874 | 61,119,429 (73.66%) | 51,081,554 (61.56%) | 10,037,875 (12.10%) | 27,130,408 (32.70%) | 27,169,628 (32.75%) |
| T06 | 71,136,968 | 51,646,052 (72.60%) | 44,691,300 (62.82%) | 6,954,752 (9.78%) | 23,618,534 (33.20%) | 23,649,512 (33.25%) |

Note: T01, T02, and T03: bud samples from three independent plants in the zws-ms population at the budding stage; T04, T05, and T06: bud samples from three independent plants in the zws-217 population at the budding stage.
