# Supplementary material for: Identification of genomic regions associated with multi-silique trait in Brassica napus
Source: BMC Genomics. 2019 Apr 23;20:304. doi: 10.1186/s12864-019-5675-4 (PMC6480887; doi:10.1186/s12864-019-5675-4)
Supplement: Supplementary file 8 — Table S7. GO annotations of the 104 genes with non-synonymous mutation SNPs in the associated regions. (DOCX 34 kb) [file 12864_2019_5675_MOESM8_ESM.docx]

Additional file 8: Table S7. GO annotations of the 104 genes with non-synonymous mutation SNPs in the associated regions

|  | Gene ID | GO Annotation |
| --- | --- | --- |
| 1 | BnaA09g46490D | Molecular Function: ATP binding (GO:0005524); Biological Process: defense response (GO:0006952); Molecular Function: nucleoside-triphosphatase activity (GO:0017111); Molecular Function: ADP binding (GO:0043531); |
| 2 | BnaA09g48390D | Molecular Function: aminoacyl-tRNA editing activity (GO:0002161); Molecular Function: leucine-tRNA ligase activity (GO:0004823); Molecular Function: ATP binding (GO:0005524); Cellular Component: cytosol (GO:0005829); Biological Process: leucyl-tRNA aminoacylation (GO:0006429); Biological Process: regulation of translational fidelity (GO:0006450); Biological Process: spermidine biosynthetic process (GO:0008295); Cellular Component: plasmodesma (GO:0009506); Cellular Component: chloroplast (GO:0009507); |
| 3 | BnaA09g45300D | Molecular Function: serine-type carboxypeptidase activity (GO:0004185); Cellular Component: extracellular region (GO:0005576); Cellular Component: vacuole (GO:0005773); Biological Process: proteolysis (GO:0006508); |
| 4 | BnaA09g46570D | Molecular Function: binding (GO:0005488); Biological Process: tryptophan catabolic process (GO:0006569); Biological Process: protein targeting to membrane (GO:0006612); Cellular Component: plasmodesma (GO:0009506); Biological Process: indoleacetic acid biosynthetic process (GO:0009684); Biological Process: positive regulation of flavonoid biosynthetic process (GO:0009963); Biological Process: response to nitrate (GO:0010167); Biological Process: regulation of plant-type hypersensitive response (GO:0010363); Biological Process: nitrate transport (GO:0015706); Molecular Function: oxidoreductase activity (GO:0016491); Biological Process: cellular cation homeostasis (GO:0030003); Biological Process: divalent metal ion transport (GO:0070838); |
| 5 | BnaC08g36210D | Molecular Function: structural molecule activity (GO:0005198); Cellular Component: nucleus (GO:0005634); Cellular Component: cytosol (GO:0005829); Biological Process: N-terminal protein myristoylation (GO:0006499); Biological Process: intracellular protein transport (GO:0006886); Biological Process: ER to Golgi vesicle-mediated transport (GO:0006888); Biological Process: Golgi organization (GO:0007030); Biological Process: vacuole organization (GO:0007033); Cellular Component: clathrin coat of trans-Golgi network vesicle (GO:0030130); Cellular Component: clathrin coat of coated pit (GO:0030132); Biological Process: amino acid import (GO:0043090); |
| 6 | BnaA09g48840D | Biological Process: glycerol ether metabolic process (GO:0006662); Molecular Function: electron carrier activity (GO:0009055); Cellular Component: chloroplast stroma (GO:0009570); Molecular Function: protein disulfide oxidoreductase activity (GO:0015035); Molecular Function: oxidoreductase activity, acting on a sulfur group of donors, disulfide as acceptor (GO:0016671); Cellular Component: chloroplast membrane (GO:0031969); Biological Process: cell redox homeostasis (GO:0045454); |
| 7 | BnaA09g45710D | Cellular Component: nucleus (GO:0005634); Biological Process: protein folding (GO:0006457); Molecular Function: zinc ion binding (GO:0008270); Biological Process: response to heat (GO:0009408); Biological Process: response to high light intensity (GO:0009644); Biological Process: response to endoplasmic reticulum stress (GO:0034976); Biological Process: response to hydrogen peroxide (GO:0042542); |
| 8 | BnaC08g35520D | Cellular Component: nucleus (GO:0005634); Biological Process: fatty acid catabolic process (GO:0009062); |
| 9 | BnaC08g37560D | Cellular Component: mitochondrion (GO:0005739); Cellular Component: endoplasmic reticulum (GO:0005783); Biological Process: vesicle-mediated transport (GO:0016192); |
| 10 | BnaA09g47300D | Molecular Function: protein binding (GO:0005515); Cellular Component: nucleus (GO:0005634); Cellular Component: plasma membrane (GO:0005886); Biological Process: N-terminal protein myristoylation (GO:0006499); Biological Process: plant-type hypersensitive response (GO:0009626); Biological Process: defense response to bacterium, incompatible interaction (GO:0009816); Molecular Function: signaling receptor activity (GO:0038023); Molecular Function: ADP binding (GO:0043531); |
| 11 | BnaA09g45600D | Cellular Component: nucleus (GO:0005634); Biological Process: response to symbiotic fungus (GO:0009610); Biological Process: cellular response to phosphate starvation (GO:0016036); Biological Process: galactolipid biosynthetic process (GO:0019375); Biological Process: cellular response to water deprivation (GO:0042631); |
| 12 | BnaA09g44900D | Biological Process: MAPK cascade (GO:0000165); Biological Process: response to cold (GO:0009409); Cellular Component: chloroplast (GO:0009507); Biological Process: salicylic acid biosynthetic process (GO:0009697); Biological Process: systemic acquired resistance, salicylic acid mediated signaling pathway (GO:0009862); Biological Process: regulation of hydrogen peroxide metabolic process (GO:0010310); Biological Process: negative regulation of defense response (GO:0031348); Biological Process: defense response to bacterium (GO:0042742); Molecular Function: ADP binding (GO:0043531); Biological Process: regulation of innate immune response (GO:0045088); Biological Process: borate transport (GO:0046713); Biological Process: defense response to fungus (GO:0050832); |
| 13 | BnaA09g49310D | Cellular Component: mitochondrion (GO:0005739); Biological Process: response to cadmium ion (GO:0046686); |
| 14 | BnaA09g42560D | Molecular Function: dicarboxylic acid transmembrane transporter activity (GO:0005310); Cellular Component: mitochondrial inner membrane (GO:0005743); Biological Process: dicarboxylic acid transport (GO:0006835); Biological Process: mitochondrial transport (GO:0006839); Biological Process: response to chitin (GO:0010200); Biological Process: proton transport (GO:0015992); Cellular Component: integral component of membrane (GO:0016021); Molecular Function: oxidative phosphorylation uncoupler activity (GO:0017077); Biological Process: transmembrane transport (GO:0055085); |
| 15 | BnaC08g42370D | Cellular Component: mitochondrion (GO:0005739); Cellular Component: plasma membrane (GO:0005886); |
| 16 | BnaA09g45760D | Cellular Component: plasma membrane (GO:0005886); Cellular Component: integral component of membrane (GO:0016021); |
| 17 | BnaA09g48150D | Molecular Function: nucleic acid binding (GO:0003676); Molecular Function: sequence-specific DNA binding transcription factor activity (GO:0003700); Cellular Component: nucleus (GO:0005634); Biological Process: regulation of transcription, DNA-templated (GO:0006355); Molecular Function: zinc ion binding (GO:0008270); Biological Process: cytokinin-activated signaling pathway (GO:0009736); Biological Process: gibberellic acid mediated signaling pathway (GO:0009740); Biological Process: trichome differentiation (GO:0010026); Biological Process: xylem development (GO:0010089); Biological Process: cell wall macromolecule metabolic process (GO:0044036); Biological Process: regulation of timing of transition from vegetative to reproductive phase (GO:0048510); |
| 18 | BnaA09g44540D | Molecular Function: calcium ion binding (GO:0005509); Cellular Component: extracellular region (GO:0005576); Biological Process: aging (GO:0007568); Cellular Component: integral component of membrane (GO:0016021); |
| 19 | BnaA09g42680D | Molecular Function: nucleotide binding (GO:0000166); |
| 20 | BnaC08g38680D | Cellular Component: mitochondrion (GO:0005739); |
| 21 | BnaA09g42650D | Molecular Function: peroxidase activity (GO:0004601); Cellular Component: extracellular region (GO:0005576); Cellular Component: cytosol (GO:0005829); Biological Process: response to oxidative stress (GO:0006979); Biological Process: determination of bilateral symmetry (GO:0009855); Biological Process: polarity specification of adaxial/abaxial axis (GO:0009944); Biological Process: meristem initiation (GO:0010014); Biological Process: regulation of meristem growth (GO:0010075); Molecular Function: heme binding (GO:0020037); Molecular Function: metal ion binding (GO:0046872); Biological Process: oxidation-reduction process (GO:0055114); |
| 22 | BnaA09g48620D | Molecular Function: sequence-specific DNA binding transcription factor activity (GO:0003700); Cellular Component: cytosol (GO:0005829); Biological Process: regulation of transcription, DNA-template (GO:0006355); Cellular Component: CCAAT-binding factor complex (GO:0016602); Molecular Function: sequence-specific DNA binding (GO:0043565); Molecular Function: protein heterodimerization activity (GO:0046982); |
| 23 | BnaA09g45080D | Molecular Function: glutathione transferase activity (GO:0004364); Cellular Component: cytoplasm (GO:0005737); Biological Process: toxin catabolic process (GO:0009407); Biological Process: response to cyclopentenone (GO:0010583); |
| 24 | BnaA09g48630D | Molecular Function: calcium:sodium antiporter activity (GO:0005432); Cellular Component: plasma membrane (GO:0005886); Biological Process: high-affinity potassium ion import (GO:0010163); Molecular Function: potassium ion transmembrane transporter activity (GO:0015079); Cellular Component: integral component of membrane (GO:0016021); Cellular Component: nuclear periphery (GO:0034399); Biological Process: sodium ion transmembrane transport (GO:0035725); |
| 25 | BnaA09g47170D | Molecular Function: nucleic acid binding (GO:0003676); Cellular Component: nucleus (GO:0005634); Cellular Component: chloroplast (GO:0009507); |
| 26 | BnaA09g44390D | Biological Process: iron ion transport (GO:0006826); Biological Process: cellular response to iron ion starvation (GO:0010106); Biological Process: response to nitrate (GO:0010167); Biological Process: nitrate transport (GO:0015706); Molecular Function: manganese ion binding (GO:0030145); Molecular Function: nutrient reservoir activity (GO:0045735); Cellular Component: apoplast (GO:0048046); |
| 27 | BnaA09g43610D | Molecular Function: structural molecule activity (GO:0005198); Cellular Component: nucleus (GO:0005634); Cellular Component: cytosol (GO:0005829); Biological Process: N-terminal protein myristoylation (GO:0006499); Biological Process: intracellular protein transport (GO:0006886); Biological Process: ER to Golgi vesicle-mediated transport (GO:0006888); Biological Process: Golgi organization (GO:0007030); Biological Process: vacuole organization (GO:0007033); Cellular Component: clathrin coat of trans-Golgi network vesicle (GO:0030130); Cellular Component: clathrin coat of coated pit (GO:0030132); Biological Process: amino acid import (GO:0043090); |
| 28 | BnaA09g46620D | Molecular Function: monooxygenase activity (GO:0004497); Molecular Function: iron ion binding (GO:0005506); Molecular Function: electron carrier activity (GO:0009055); Cellular Component: plasmodesma (GO:0009506); Biological Process: response to nitrate (GO:0010167); Biological Process: nitrate transport (GO:0015706); Molecular Function: oxidoreductase activity, acting on paired donors, with incorporation or reduction of molecular oxygen (GO:0016705); Molecular Function: oxygen binding (GO:0019825); Molecular Function: heme binding (GO:0020037); Biological Process: oxidation-reduction process (GO:0055114); |
| 29 | BnaA09g42170D | Cellular Component: plasma membrane (GO:0005886); Cellular Component: anchored component of membrane (GO:0031225); |
| 30 | BnaA09g43250D | Cellular Component: mitochondrion (GO:0005739); |
| 31 | BnaA09g49380D | Biological Process: nuclear-transcribed mRNA catabolic process (GO:0000956); Biological Process: respiratory burst involved in defense response (GO:0002679); Molecular Function: sequence-specific DNA binding transcription factor activity (GO:0003700); Cellular Component: nucleus (GO:0005634); Cellular Component: cytosol (GO:0005829); Biological Process: protein glycosylation (GO:0006486); Biological Process: response to xenobiotic stimulus (GO:0009410); Biological Process: response to chitin (GO:0010200); Biological Process: intracellular signal transduction (GO:0035556); Biological Process: positive regulation of transcription, DNA-templated (GO:0045893); |
| 32 | BnaA09g48400D | Cellular Component: nucleus (GO:0005634); Cellular Component: plasma membrane (GO:0005886); Biological Process: epidermal cell fate specification (GO:0009957); Molecular Function: kinase activity (GO:0016301); |
| 33 | BnaA09g46650D | Molecular Function: monooxygenase activity (GO:0004497); Molecular Function: iron ion binding (GO:0005506); Molecular Function: electron carrier activity (GO:0009055); Cellular Component: plasmodesma (GO:0009506); Biological Process: response to nitrate (GO:0010167); Biological Process: nitrate transport (GO:0015706); Molecular Function: oxidoreductase activity, acting on paired donors, with incorporation or reduction of molecular oxygen (GO:0016705); Molecular Function: oxygen binding (GO:0019825); Molecular Function: heme binding (GO:0020037); Biological Process: oxidation-reduction process (GO:0055114); |
| 34 | BnaA09g47940D | Cellular Component: nucleus (GO:0005634); |
| 35 | BnaA09g46190D | Cellular Component: extracellular region (GO:0005576); Molecular Function: growth factor activity (GO:0008083); Biological Process: cell proliferation (GO:0008283); Biological Process: cell growth (GO:0016049); Cellular Component: extracellular matrix (GO:0031012); |
| 36 | BnaA09g45130D | Cellular Component: nucleus (GO:0005634); Cellular Component: plasma membrane (GO:0005886); Biological Process: pattern specification process (GO:0007389); Biological Process: anatomical structure morphogenesis (GO:0009653); Biological Process: auxin metabolic process (GO:0009850); Biological Process: gene silencing (GO:0016458); Biological Process: regulation of gene expression, epigenetic (GO:0040029); Biological Process: single-organism developmental process (GO:0044767); Biological Process: system development (GO:0048731); Cellular Component: pollen tube tip (GO:0090404); Biological Process: regulation of auxin polar transport (GO:2000012); |
| 37 | BnaA09g45100D | Molecular Function: ribokinase activity (GO:0004747); Biological Process: D-ribose metabolic process (GO:0006014); Cellular Component: chloroplast stroma (GO:0009570); Biological Process: phosphorylation (GO:0016310); |
| 38 | BnaA09g42850D | Cellular Component: mitochondrion (GO:0005739); |
| 39 | BnaA09g48060D | Molecular Function: sequence-specific DNA binding transcription factor activity (GO:0003700); Cellular Component: nucleus (GO:0005634); Cellular Component: cytoplasm (GO:0005737); Biological Process: regulation of transcription, DNA-templated (GO:0006355); Biological Process: amino acid transport (GO:0006865); Molecular Function: protein dimerization activity (GO:0046983); |
| 40 | BnaA09g49100D | Molecular Function: sequence-specific DNA binding transcription factor activity (GO:0003700); Cellular Component: nucleus (GO:0005634); |
| 41 | BnaA09g43100D | Cellular Component: Golgi apparatus (GO:0005794); Biological Process: transport (GO:0006810); Molecular Function: phosphatidylinositol transporter activity (GO:0008526); Biological Process: flower development (GO:0009908); |
| 42 | BnaA09g45860D | Biological Process: protein targeting to mitochondrion (GO:0006626); |
| 43 | BnaA09g43150D | Cellular Component: nucleus (GO:0005634); Biological Process: response to stress (GO:0006950); Biological Process: response to water (GO:0009415); Biological Process: embryo development ending in seed dormancy (GO:0009793); Biological Process: terpenoid biosynthetic process (GO:0016114); |
| 44 | BnaA09g44990D | Cellular Component: nucleus (GO:0005634); Biological Process: photoperiodism (GO:0009648); |
| 45 | BnaA09g45720D | Cellular Component: nucleus (GO:0005634); Biological Process: alcohol metabolic process (GO:0006066); Molecular Function: choline dehydrogenase activity (GO:0008812); Cellular Component: chloroplast (GO:0009507); Molecular Function: aldehyde-lyase activity (GO:0016832); Molecular Function: flavin adenine dinucleotide binding (GO:0050660); Biological Process: oxidation-reduction process (GO:0055114); |
| 46 | BnaA09g48950D | Molecular Function: nucleic acid binding (GO:0003676); Molecular Function: sequence-specific DNA binding transcription factor activity (GO:0003700); Cellular Component: nuclear envelope (GO:0005635); Biological Process: regulation of transcription, DNA-templated (GO:0006355); Biological Process: nucleocytoplasmic transport (GO:0006913); Biological Process: nucleus organization (GO:0006997); Molecular Function: zinc ion binding (GO:0008270); Biological Process: lateral root development (GO:0048527); |
| 47 | BnaA09g44360D | Biological Process: response to oxidative stress (GO:0006979); Biological Process: response to wounding (GO:0009611); Biological Process: response to insect (GO:0009625); Biological Process: systemic acquired resistance, salicylic acid mediated signaling pathway (GO:0009862); Biological Process: response to chitin (GO:0010200); Biological Process: endoplasmic reticulum unfolded protein response (GO:0030968); Biological Process: negative regulation of defense response (GO:0031348); Biological Process: defense response to fungus (GO:0050832); |
| 48 | BnaA09g47570D | Cellular Component: cytoplasm (GO:0005737); |
| 49 | BnaA09g45970D | Molecular Function: acid phosphatase activity (GO:0003993); Molecular Function: protein serine/threonine phosphatase activity (GO:0004722); Cellular Component: extracellular region (GO:0005576); Cellular Component: mitochondrion (GO:0005739); Cellular Component: endosome (GO:0005768); Cellular Component: Golgi apparatus (GO:0005794); Cellular Component: trans-Golgi network (GO:0005802); Biological Process: regulation of carbohydrate metabolic process (GO:0006109); Cellular Component: chloroplast (GO:0009507); Molecular Function: metal ion binding (GO:0046872); |
| 50 | BnaA09g46720D | Molecular Function: protein binding (GO:0005515); Cellular Component: nucleus (GO:0005634); Cellular Component: cytosol (GO:0005829); Biological Process: response to cytokinin (GO:0009735); Biological Process: embryonic pattern specification (GO:0009880); Biological Process: specification of organ axis polarity (GO:0010084); Biological Process: cotyledon development (GO:0048825); |
| 51 | BnaA09g48070D | Molecular Function: sequence-specific DNA binding transcription factor activity (GO:0003700); Cellular Component: nucleus (GO:0005634); Cellular Component: cytoplasm (GO:0005737); Biological Process: regulation of transcription, DNA-templated (GO:0006355); Biological Process: amino acid transport (GO:0006865); Molecular Function: protein dimerization activity (GO:0046983); |
| 52 | BnaA09g45000D | Biological Process: RNA splicing, via endonucleolytic cleavage and ligation (GO:0000394); Cellular Component: nucleus (GO:0005634); Biological Process: transcription from RNA polymerase II promoter (GO:0006366); Biological Process: cytokinin-activated signaling pathway (GO:0009736); Biological Process: jasmonic acid mediated signaling pathway (GO:0009867); Biological Process: regulation of ethylene-activated signaling pathway (GO:0010104); |
| 53 | BnaA09g47600D | Cellular Component: mitochondrion (GO:0005739); |
| 54 | BnaC08g36330D | Biological Process: MAPK cascade (GO:0000165); Molecular Function: chromatin binding (GO:0003682); Molecular Function: sequence-specific DNA binding transcription factor activity (GO:0003700); Cellular Component: nucleus (GO:0005634); Biological Process: rRNA processing (GO:0006364); Biological Process: protein targeting to membrane (GO:0006612); Biological Process: response to bacterium (GO:0009617); Biological Process: systemic acquired resistance, salicylic acid mediated signaling pathway (GO:0009862); Biological Process: jasmonic acid mediated signaling pathway (GO:0009867); Biological Process: chloroplast relocation (GO:0009902); Biological Process: negative regulation of flower development (GO:0009910); Biological Process: leaf morphogenesis (GO:0009965); Biological Process: thylakoid membrane organization (GO:0010027); Biological Process: photosystem II assembly (GO:0010207); Biological Process: regulation of hydrogen peroxide metabolic process (GO:0010310); Biological Process: regulation of plant-type hypersensitive response (GO:0010363); Biological Process: regulation of chlorophyll biosynthetic process (GO:0010380); Biological Process: cell differentiation (GO:0030154); Biological Process: negative regulation of defense response (GO:0031348); Biological Process: regulation of protein dephosphorylation (GO:0035304); Biological Process: transcription from plastid promoter (GO:0042793); Molecular Function: transcription regulatory region DNA binding (GO:0044212); Biological Process: positive regulation of transcription, DNA-templated (GO:0045893); |
| 55 | BnaA09g46480D | Molecular Function: ATP binding (GO:0005524); Biological Process: N-terminal protein myristoylation (GO:0006499); Biological Process: defense response (GO:0006952); Molecular Function: nucleoside-triphosphatase activity (GO:0017111); Molecular Function: ADP binding (GO:0043531); |
| 56 | BnaA09g43560D | Cellular Component: plasma membrane (GO:0005886); Biological Process: regulation of anthocyanin biosynthetic process (GO:0031540); |
| 57 | BnaC08g35230D | Cellular Component: nucleus (GO:0005634); Biological Process: DNA repair (GO:0006281); Molecular Function: oxidoreductase activity, acting on single donors with incorporation of molecular oxygen, incorporation of two atoms of oxygen (GO:0016702); Molecular Function: oxidoreductase activity, acting on paired donors, with incorporation or reduction of molecular oxygen, 2-oxoglutarate as one donor, and incorporation of one atom each of oxygen into both donors (GO:0016706); Molecular Function: DNA demethylase activity (GO:0035514); Biological Process: oxidation-reduction process (GO:0055114); Biological Process: DNA demethylation (GO:0080111); |
| 58 | BnaA09g46470D | Molecular Function: nucleotide binding (GO:0000166); Molecular Function: protein binding (GO:0005515); Cellular Component: intracellular (GO:0005622); Cellular Component: plasma membrane (GO:0005886); Biological Process: plant-type hypersensitive response (GO:0009626); Biological Process: defense response to bacterium, incompatible interaction (GO:0009816); Molecular Function: signaling receptor activity (GO:0038023); |
| 59 | BnaA09g43490D | Biological Process: single-organism process (GO:0044699); |
| 60 | BnaA09g42750D | Cellular Component: nucleus (GO:0005634); Biological Process: DNA repair (GO:0006281); Molecular Function: oxidoreductase activity, acting on single donors with incorporation of molecular oxygen, incorporation of two atoms of oxygen (GO:0016702); Molecular Function: oxidoreductase activity, acting on paired donors, with incorporation or reduction of molecular oxygen, 2-oxoglutarate as one donor, and incorporation of one atom each of oxygen into both donors (GO:0016706); Molecular Function: DNA demethylase activity (GO:0035514); Biological Process: oxidation-reduction process (GO:0055114); Biological Process: DNA demethylation (GO:0080111); |
| 61 | BnaC08g39110D | Molecular Function: serine-type carboxypeptidase activity (GO:0004185); Cellular Component: extracellular region (GO:0005576); Cellular Component: vacuole (GO:0005773); Biological Process: proteolysis (GO:0006508); |
| 62 | BnaA09g42920D | Cellular Component: extracellular region (GO:0005576); Cellular Component: vesicle (GO:0031982); Biological Process: regulation of double fertilization forming a zygote and endosperm (GO:0080155); Biological Process: regulation of protein localization to cell surface (GO:2000008); |
| 63 | BnaC08g40310D | Biological Process: MAPK cascade (GO:0000165); Biological Process: salicylic acid biosynthetic process (GO:0009697); Biological Process: systemic acquired resistance, salicylic acid mediated signaling pathway (GO:0009862); Biological Process: regulation of hydrogen peroxide metabolic process (GO:0010310); Biological Process: negative regulation of defense response (GO:0031348); Biological Process: defense response to bacterium (GO:0042742); Biological Process: regulation of innate immune response (GO:0045088); Biological Process: defense response to fungus (GO:0050832); |
| 64 | BnaA09g44210D | Molecular Function: DNA binding (GO:0003677); Molecular Function: sequence-specific DNA binding transcription factor activity (GO:0003700); Molecular Function: protein binding (GO:0005515); Cellular Component: nucleus (GO:0005634); Cellular Component: cytosol (GO:0005829); Biological Process: brassinosteroid mediated signaling pathway (GO:0009742); Biological Process: negative regulation of transcription, DNA-templated (GO:0045892); Biological Process: seed development (GO:0048316); Biological Process: ovule development (GO:0048481); |
| 65 | BnaA09g46820D | Cellular Component: nucleus (GO:0005634); Biological Process: protein targeting to mitochondrion (GO:0006626); Biological Process: embryo sac egg cell differentiation (GO:0009560); |
| 66 | BnaA09g47910D | Molecular Function: protein serine/threonine kinase activity (GO:0004674); Molecular Function: ATP binding (GO:0005524); Cellular Component: plasma membrane (GO:0005886); Biological Process: protein phosphorylation (GO:0006468); Biological Process: ethylene biosynthetic process (GO:0009693); Biological Process: response to ethylene (GO:0009723); Biological Process: abscisic acid-activated signaling pathway (GO:0009738); Biological Process: intracellular signal transduction (GO:0035556); |
| 67 | BnaA09g43370D | Biological Process: phosphatidylinositol biosynthetic process (GO:0006661); |
| 68 | BnaA09g49620D | Molecular Function: DNA binding (GO:0003677); Cellular Component: nucleus (GO:0005634); Biological Process: toxin catabolic process (GO:0009407); Biological Process: response to cyclopentenone (GO:0010583); Biological Process: negative regulation of transcription, DNA-templated (GO:0045892); Biological Process: organ development (GO:0048513); Biological Process: regulation of secondary cell wall biogenesis (GO:2000652); |
| 69 | BnaA09g44160D | Biological Process: very long-chain fatty acid metabolic process (GO:0000038); Cellular Component: nucleus (GO:0005634); Biological Process: fatty acid biosynthetic process (GO:0006633); Biological Process: response to cold (GO:0009409); Cellular Component: membrane (GO:0016020); Molecular Function: transferase activity, transferring acyl groups other than amino-acyl groups (GO:0016747); Biological Process: cuticle development (GO:0042335); |
| 70 | BnaA09g45270D | Biological Process: glutamine metabolic process (GO:0006541); Molecular Function: hydrolase activity (GO:0016787); Biological Process: regulation of secondary shoot formation (GO:2000032); |
| 71 | BnaA09g45750D | Molecular Function: RNA binding (GO:0003723); Cellular Component: nucleus (GO:0005634); Biological Process: response to abscisic acid (GO:0009737); |
| 72 | BnaA09g45560D | Molecular Function: DNA binding (GO:0003677); Molecular Function: sequence-specific DNA binding transcription factor activity (GO:0003700); Cellular Component: nucleus (GO:0005634); Biological Process: regulation of transcription, DNA-templated (GO:0006355); Biological Process: response to ethylene (GO:0009723); Biological Process: regulation of developmental process (GO:0050793); |
| 73 | BnaA09g42840D | Molecular Function: nucleotide binding (GO:0000166); Biological Process: mRNA splicing, via spliceosome (GO:0000398); Cellular Component: nucleus (GO:0005634); Molecular Function: AU-rich element binding (GO:0017091); Biological Process: mRNA stabilization (GO:0048255); |
| 74 | BnaA09g45070D | Molecular Function: glutathione transferase activity (GO:0004364); Cellular Component: cytoplasm (GO:0005737); Biological Process: toxin catabolic process (GO:0009407); Biological Process: response to cyclopentenone (GO:0010583); |
| 75 | BnaA09g44010D | Molecular Function: nucleic acid binding (GO:0003676); Cellular Component: nucleus (GO:0005634); |
| 76 | BnaA09g45640D | Biological Process: transition metal ion transport (GO:0000041); Molecular Function: DNA binding (GO:0003677); Molecular Function: sequence-specific DNA binding transcription factor activity (GO:0003700); Molecular Function: histidine-tRNA ligase activity (GO:0004821); Cellular Component: nucleus (GO:0005634); Cellular Component: mitochondrion (GO:0005739); Biological Process: regulation of transcription, DNA-templated (GO:0006355); Biological Process: histidyl-tRNA aminoacylation (GO:0006427); Cellular Component: chloroplast (GO:0009507); Biological Process: cellular response to phosphate starvation (GO:0016036); Biological Process: primary root development (GO:0080022); |
| 77 | BnaA09g47440D | Cellular Component: vacuolar membrane (GO:0005774); Cellular Component: Golgi apparatus (GO:0005794); Cellular Component: cytosol (GO:0005829); Cellular Component: plasma membrane (GO:0005886); Cellular Component: plant-type cell wall (GO:0009505); Molecular Function: carbohydrate binding (GO:0030246); Cellular Component: apoplast (GO:0048046); |
| 78 | BnaA09g45030D | Biological Process: response to hypoxia (GO:0001666); Molecular Function: L-alanine:2-oxoglutarate aminotransferase activity (GO:0004021); Molecular Function: ATP binding (GO:0005524); Cellular Component: mitochondrion (GO:0005739); Cellular Component: cytosol (GO:0005829); Cellular Component: chloroplast (GO:0009507); Molecular Function: 1-aminocyclopropane-1-carboxylate synthase activity (GO:0016847); Biological Process: L-alanine catabolic process, by transamination (GO:0019481); Molecular Function: pyridoxal phosphate binding (GO:0030170); Biological Process: 1-aminocyclopropane-1-carboxylate biosynthetic process (GO:0042218); Biological Process: response to cadmium ion (GO:0046686); |
| 79 | BnaA09g49290D | Biological Process: MAPK cascade (GO:0000165); Biological Process: regionalization (GO:0003002); Molecular Function: DNA binding (GO:0003677); Molecular Function: sequence-specific DNA binding transcription factor activity (GO:0003700); Cellular Component: nucleus (GO:0005634); Biological Process: regulation of transcription, DNA-templated (GO:0006355); Biological Process: protein targeting to membrane (GO:0006612); Biological Process: detection of biotic stimulus (GO:0009595); Biological Process: response to wounding (GO:0009611); Biological Process: response to insect (GO:0009625); Biological Process: salicylic acid biosynthetic process (GO:0009697); Biological Process: systemic acquired resistance, salicylic acid mediated signaling pathway (GO:0009862); Biological Process: jasmonic acid mediated signaling pathway (GO:0009867); Biological Process: response to chitin (GO:0010200); Biological Process: regulation of hydrogen peroxide metabolic process (GO:0010310); Biological Process: regulation of plant-type hypersensitive response (GO:0010363); Biological Process: regulation of glucosinolate biosynthetic process (GO:0010439); Biological Process: negative regulation of defense response (GO:0031348); Biological Process: defense response to bacterium (GO:0042742); Biological Process: regulation of multi-organism process (GO:0043900); Biological Process: defense response to fungus (GO:0050832); |
| 80 | BnaA09g43780D | Cellular Component: nucleus (GO:0005634); |
| 81 | BnaA09g45150D | Biological Process: amino acid transmembrane transport (GO:0003333); Cellular Component: vacuolar membrane (GO:0005774); Cellular Component: plasma membrane (GO:0005886); Biological Process: response to brassinosteroid (GO:0009741); Molecular Function: basic amino acid transmembrane transporter activity (GO:0015174); Molecular Function: cationic amino acid transmembrane transporter activity (GO:0015326); Cellular Component: integral component of membrane (GO:0016021); |
| 82 | BnaA09g45440D | Molecular Function: nucleotide binding (GO:0000166); Molecular Function: transporter activity (GO:0005215); Cellular Component: plasma membrane (GO:0005886); Biological Process: transport (GO:0006810); Biological Process: aspartate family amino acid biosynthetic process (GO:0009067); Cellular Component: chloroplast (GO:0009507); Molecular Function: oxidoreductase activity, acting on the aldehyde or oxo group of donors, NAD or NADP as acceptor (GO:0016620); Molecular Function: coenzyme binding (GO:0050662); |
| 83 | BnaC08g38340D | Cellular Component: nucleus (GO:0005634); Biological Process: xylem development (GO:0010089); Biological Process: cell wall macromolecule metabolic process (GO:0044036); |
| 84 | BnaA09g45820D | Cellular Component: Golgi apparatus (GO:0005794); Molecular Function: fucosyltransferase activity (GO:0008417); |
| 85 | BnaA09g42580D | Molecular Function: 6-phosphofructokinase activity (GO:0003872); Molecular Function: ATP binding (GO:0005524); Cellular Component: 6-phosphofructokinase complex (GO:0005945); Biological Process: fructose 6-phosphate metabolic process (GO:0006002); Biological Process: gluconeogenesis (GO:0006094); Biological Process: glycolysis (GO:0006096); Biological Process: cytoskeleton organization (GO:0007010); Cellular Component: chloroplast (GO:0009507); Biological Process: proteasomal protein catabolic process (GO:0010498); Biological Process: phosphorylation (GO:0016310); |
| 86 | BnaA09g43730D | Cellular Component: nucleus (GO:0005634); |
| 87 | BnaA09g47560D | Cellular Component: cytoplasm (GO:0005737); |
| 88 | BnaA09g46850D | Molecular Function: ubiquitin-protein ligase activity (GO:0004842); Cellular Component: nucleus (GO:0005634); Molecular Function: zinc ion binding (GO:0008270); Cellular Component: chloroplast (GO:0009507); Biological Process: protein ubiquitination (GO:0016567); |
| 89 | BnaA09g46580D | Molecular Function: monooxygenase activity (GO:0004497); Molecular Function: iron ion binding (GO:0005506); Biological Process: tryptophan catabolic process (GO:0006569); Biological Process: protein targeting to membrane (GO:0006612); Molecular Function: electron carrier activity (GO:0009055); Biological Process: indoleacetic acid biosynthetic process (GO:0009684); Biological Process: positive regulation of flavonoid biosynthetic process (GO:0009963); Biological Process: response to nitrate (GO:0010167); Biological Process: regulation of plant-type hypersensitive response (GO:0010363); Biological Process: nitrate transport (GO:0015706); Molecular Function: oxidoreductase activity, acting on paired donors, with incorporation or reduction of molecular oxygen (GO:0016705); Molecular Function: oxygen binding (GO:0019825); Molecular Function: heme binding (GO:0020037); Biological Process: cellular cation homeostasis (GO:0030003); Biological Process: oxidation-reduction process (GO:0055114); Biological Process: divalent metal ion transport (GO:0070838); |
| 90 | BnaA09g46300D | Molecular Function: DNA binding (GO:0003677); Cellular Component: nucleus (GO:0005634); Cellular Component: Golgi apparatus (GO:0005794); Cellular Component: cytosol (GO:0005829); Biological Process: glucose catabolic process (GO:0006007); Molecular Function: ARF GTPase activator activity (GO:0008060); Molecular Function: zinc ion binding (GO:0008270); Biological Process: cellulose biosynthetic process (GO:0030244); Biological Process: regulation of ARF GTPase activity (GO:0032312); Biological Process: Golgi vesicle transport (GO:0048193); |
| 91 | BnaA09g48960D | Molecular Function: prephenate dehydratase activity (GO:0004664); Cellular Component: cytosol (GO:0005829); Biological Process: tyrosine biosynthetic process (GO:0006571); Biological Process: L-phenylalanine biosynthetic process (GO:0009094); Cellular Component: chloroplast (GO:0009507); Biological Process: vernalization response (GO:0010048); Biological Process: response to low fluence blue light stimulus by blue low-fluence system (GO:0010244); Biological Process: anthocyanin accumulation in tissues in response to UV light (GO:0043481); Molecular Function: arogenate dehydratase activity (GO:0047769); Biological Process: carpel development (GO:0048440); Biological Process: response to karrikin (GO:0080167); |
| 92 | BnaC08g38270D | Molecular Function: transcription cofactor activity (GO:0003712); Molecular Function: histone acetyltransferase activity (GO:0004402); Cellular Component: nucleus (GO:0005634); Biological Process: regulation of transcription, DNA-templated (GO:0006355); Biological Process: cell adhesion (GO:0007155); Molecular Function: zinc ion binding (GO:0008270); Biological Process: embryo development ending in seed dormancy (GO:0009793); Biological Process: flower development (GO:0009908); Biological Process: trichome morphogenesis (GO:0010090); Biological Process: vegetative to reproductive phase transition of meristem (GO:0010228); Biological Process: histone acetylation (GO:0016573); Biological Process: actin nucleation (GO:0045010); Biological Process: root hair cell differentiation (GO:0048765); Biological Process: cell wall organization (GO:0071555); |
| 93 | BnaA09g47370D | Biological Process: regulation of transport (GO:0051049); |
| 94 | BnaC08g34490D | Biological Process: metabolic process (GO:0008152); Molecular Function: abscisic acid glucosyltransferase activity (GO:0010294); Molecular Function: indole-3-acetate beta-glucosyltransferase activity (GO:0047215); Molecular Function: quercetin 7-O-glucosyltransferase activity (GO:0080044); |
| 95 | BnaA09g42700D | Biological Process: regulation of vernalization response (GO:0010219); Cellular Component: mediator complex (GO:0016592); Biological Process: negative regulation of transcription, DNA-templated (GO:0045892); Biological Process: carpel development (GO:0048440); Biological Process: petal development (GO:0048441); Biological Process: sepal development (GO:0048442); Biological Process: stamen development (GO:0048443); Biological Process: regulation of timing of transition from vegetative to reproductive phase (GO:0048510); Biological Process: specification of floral organ number (GO:0048833); |
| 96 | BnaC08g42470D | Cellular Component: nucleus (GO:0005634); Biological Process: transcription, DNA-templated (GO:0006351); Molecular Function: sequence-specific DNA binding (GO:0043565); |
| 97 | BnaA09g49420D | Molecular Function: protein kinase activity (GO:0004672); Molecular Function: ATP binding (GO:0005524); Cellular Component: plasma membrane (GO:0005886); Biological Process: protein phosphorylation (GO:0006468); Molecular Function: carbohydrate binding (GO:0030246); Cellular Component: anchored component of membrane (GO:0031225); |
| 98 | BnaA09g45700D | Molecular Function: structural constituent of ribosome (GO:0003735); Cellular Component: ribosome (GO:0005840); Biological Process: translation (GO:0006412); Cellular Component: chloroplast (GO:0009507); |
| 99 | BnaA09g49340D | Cellular Component: nucleosome (GO:0000786); Molecular Function: DNA binding (GO:0003677); Cellular Component: nucleolus (GO:0005730); Cellular Component: vacuole (GO:0005773); Biological Process: nucleosome assembly (GO:0006334); Cellular Component: thylakoid (GO:0009579); Molecular Function: protein heterodimerization activity (GO:0046982); |
| 100 | BnaA09g45610D | Cellular Component: nucleus (GO:0005634); |
| 101 | BnaA09g47860D | Molecular Function: protein serine/threonine kinase activity (GO:0004674); Molecular Function: protein binding (GO:0005515); Molecular Function: ATP binding (GO:0005524); Cellular Component: nucleus (GO:0005634); Cellular Component: cytosol (GO:0005829); Biological Process: protein phosphorylation (GO:0006468); Biological Process: ubiquitin-dependent protein catabolic process (GO:0006511); Biological Process: response to salt stress (GO:0009651); Biological Process: response to abscisic acid (GO:0009737); Cellular Component: membrane (GO:0016020); Biological Process: regulation of circadian rhythm (GO:0042752); Molecular Function: phosphatidic acid binding (GO:0070300); Biological Process: primary root development (GO:0080022); |
| 102 | BnaA09g44350D | Cellular Component: cytoplasm (GO:0005737); Molecular Function: hydrolase activity (GO:0016787); |
| 103 | BnaA09g49530D | Cellular Component: nucleus (GO:0005634); |
| 104 | BnaA09g45950D | Cellular Component: nucleus (GO:0005634); |
