# Supplementary material for: Identification of genomic regions associated with multi-silique trait in Brassica napus
Source: BMC Genomics. 2019 Apr 23;20:304. doi: 10.1186/s12864-019-5675-4 (PMC6480887; doi:10.1186/s12864-019-5675-4)
Supplement: Supplementary file 9 — Table S8. GO annotations of the 19 genes with frame-shift mutation InDels in the associated regions. (DOCX 17 kb) [file 12864_2019_5675_MOESM9_ESM.docx]

Additional file 9: Table S8. GO annotations of the 19 genes with frame-shift mutation InDels in the associated regions

|  | Gene ID | GO Annotation |
| --- | --- | --- |
| 1 | BnaA09g45300D | Molecular Function: serine-type carboxypeptidase activity (GO:0004185); Cellular Component: extracellular region (GO:0005576); Cellular Component: vacuole (GO:0005773); Biological Process: proteolysis (GO:0006508); |
| 2 | BnaC08g36210D | Molecular Function: structural molecule activity (GO:0005198); Cellular Component: nucleus (GO:0005634); Cellular Component: cytosol (GO:0005829); Biological Process: N-terminal protein myristoylation (GO:0006499); Biological Process: intracellular protein transport (GO:0006886); Biological Process: ER to Golgi vesicle-mediated transport (GO:0006888); Biological Process: Golgi organization (GO:0007030); Biological Process: vacuole organization (GO:0007033); Cellular Component: clathrin coat of trans-Golgi network vesicle (GO:0030130); Cellular Component: clathrin coat of coated pit (GO:0030132); Biological Process: amino acid import (GO:0043090); |
| 3 | BnaC08g37560D | Cellular Component: mitochondrion (GO:0005739); Cellular Component: endoplasmic reticulum (GO:0005783); Biological Process: vesicle-mediated transport (GO:0016192); |
| 4 | BnaA09g47300D | Molecular Function: protein binding (GO:0005515); Cellular Component: nucleus (GO:0005634); Cellular Component: plasma membrane (GO:0005886); Biological Process: N-terminal protein myristoylation (GO:0006499); Biological Process: plant-type hypersensitive response (GO:0009626); Biological Process: defense response to bacterium, incompatible interaction (GO:0009816); Molecular Function: signaling receptor activity (GO:0038023); Molecular Function: ADP binding (GO:0043531); |
| 5 | BnaA09g45600D | Cellular Component: nucleus (GO:0005634); Biological Process: response to symbiotic fungus (GO:0009610); Biological Process: cellular response to phosphate starvation (GO:0016036); Biological Process: galactolipid biosynthetic process (GO:0019375); Biological Process: cellular response to water deprivation (GO:0042631); |
| 6 | BnaA09g47170D | Molecular Function: nucleic acid binding (GO:0003676); Cellular Component: nucleus (GO:0005634); Cellular Component: chloroplast (GO:0009507); |
| 7 | BnaA09g42850D | Cellular Component: mitochondrion (GO:0005739); |
| 8 | BnaA09g46720D | Molecular Function: protein binding (GO:0005515); Cellular Component: nucleus (GO:0005634); Cellular Component: cytosol (GO:0005829); Biological Process: response to cytokinin (GO:0009735); Biological Process: embryonic pattern specification (GO:0009880); Biological Process: specification of organ axis polarity (GO:0010084); Biological Process: cotyledon development (GO:0048825); |
| 9 | BnaA09g43990D | Molecular Function: actin binding (GO:0003779); Cellular Component: cell wall (GO:0005618); Cellular Component: nucleolus (GO:0005730); Cellular Component: spindle (GO:0005819); Cellular Component: cytosol (GO:0005829); Cellular Component: plasma membrane (GO:0005886); Biological Process: actin polymerization or depolymerization (GO:0008154); Cellular Component: plasmodesma (GO:0009506); Cellular Component: chloroplast (GO:0009507); Cellular Component: phragmoplast (GO:0009524); Biological Process: unidimensional cell growth (GO:0009826); Cellular Component: actin cytoskeleton (GO:0015629); Cellular Component: apoplast (GO:0048046); |
| 10 | BnaA09g43560D | Cellular Component: plasma membrane (GO:0005886); Biological Process: regulation of anthocyanin biosynthetic process (GO:0031540); |
| 11 | BnaA09g42750D | Cellular Component: nucleus (GO:0005634); Biological Process: DNA repair (GO:0006281); Molecular Function: oxidoreductase activity, acting on single donors with incorporation of molecular oxygen, incorporation of two atoms of oxygen (GO:0016702); Molecular Function: oxidoreductase activity, acting on paired donors, with incorporation or reduction of molecular oxygen, 2-oxoglutarate as one donor, and incorporation of one atom each of oxygen into both donors (GO:0016706); Molecular Function: DNA demethylase activity (GO:0035514); Biological Process: oxidation-reduction process (GO:0055114); Biological Process: DNA demethylation (GO:0080111); |
| 12 | BnaA09g44210D | Molecular Function: DNA binding (GO:0003677); Molecular Function: sequence-specific DNA binding transcription factor activity (GO:0003700); Molecular Function: protein binding (GO:0005515); Cellular Component: nucleus (GO:0005634); Cellular Component: cytosol (GO:0005829); Biological Process: brassinosteroid mediated signaling pathway (GO:0009742); Biological Process: negative regulation of transcription, DNA-templated (GO:0045892); Biological Process: seed development (GO:0048316); Biological Process: ovule development (GO:0048481); |
| 13 | BnaA09g43780D | Cellular Component: nucleus (GO:0005634); |
| 14 | BnaA09g47000D | Biological Process: mRNA splicing, via spliceosome (GO:0000398); Cellular Component: nucleus (GO:0005634); Biological Process: rRNA processing (GO:0006364); Biological Process: protein maturation (GO:0051604); |
| 15 | BnaA09g45820D | Cellular Component: Golgi apparatus (GO:0005794); Molecular Function: fucosyltransferase activity (GO:0008417); |
| 16 | BnaA09g44620D | Cellular Component: nucleus (GO:0005634); |
| 17 | BnaA09g43730D | Cellular Component: nucleus (GO:0005634); |
| 18 | BnaC08g36510D | Molecular Function: nucleotide binding (GO:0000166); Molecular Function: glucokinase activity (GO:0004340); Molecular Function: protein binding (GO:0005515); Cellular Component: nucleus (GO:0005634); Cellular Component: mitochondrion (GO:0005739); Cellular Component: vacuolar membrane (GO:0005774); Molecular Function: zinc ion binding (GO:0008270); Molecular Function: fructokinase activity (GO:0008865); Cellular Component: plastid (GO:0009536); Biological Process: response to fructose (GO:0009750); Biological Process: transpiration (GO:0010148); Biological Process: glucose mediated signaling pathway (GO:0010255); Biological Process: programmed cell death (GO:0012501); Biological Process: hexose catabolic process (GO:0019320); Biological Process: stomatal closure (GO:0090332); |
| 19 | BnaA09g45950D | Cellular Component: nucleus (GO:0005634); |
