# Supplementary material for: Identification of genomic regions associated with multi-silique trait in Brassica napus
Source: BMC Genomics. 2019 Apr 23;20:304. doi: 10.1186/s12864-019-5675-4 (PMC6480887; doi:10.1186/s12864-019-5675-4)
Supplement: Supplementary file 11 — Table S10. Information about the DEGs between zws-ms and zws-217. (DOCX 40 kb) [file 12864_2019_5675_MOESM11_ESM.docx]

Additional file 11: Table S10. Information about the 129 DEGs between zws-ms and zws-217

|  | Gene ID | Relative expression in zws-ms | GO annotation |
| --- | --- | --- | --- |
| 1 | BnaA01g10540D | down | Cellular Component: nucleus (GO:0005634); |
| 2 | BnaA01g26720D | down | Molecular Function: aspartic-type endopeptidase activity (GO:0004190); Cellular Component: extracellular region (GO:0005576); Cellular Component: endoplasmic reticulum (GO:0005783); Biological Process: proteolysis (GO:0006508); Biological Process: response to water deprivation (GO:0009414); Biological Process: response to abscisic acid (GO:0009737); |
| 3 | BnaA02g02630D | down | Cellular Component: Golgi apparatus (GO:0005794); Biological Process: pollen development (GO:0009555); Biological Process: pollen tube growth (GO:0009860); Molecular Function: polygalacturonate 4-alpha-galacturonosyltransferase activity (GO:0047262); Biological Process: cell wall pectin biosynthetic process (GO:0052325); Cellular Component: pollen tube (GO:0090406); |
| 4 | BnaA02g03080D | down | -- |
| 5 | BnaA03g35870D | up | Molecular Function: guanylate kinase activity (GO:0004385); Cellular Component: cytoplasm (GO:0005737); Biological Process: purine nucleotide metabolic process (GO:0006163); Biological Process: response to chitin (GO:0010200); Biological Process: phosphorylation (GO:0016310); Biological Process: endoplasmic reticulum unfolded protein response (GO:0030968); Biological Process: defense response to fungus (GO:0050832); |
| 6 | BnaA04g00770D | down | Molecular Function: DNA binding (GO:0003677); Cellular Component: nucleus (GO:0005634); Biological Process: stomatal complex morphogenesis (GO:0010103); |
| 7 | BnaA04g06410D | up | Biological Process: heme biosynthetic process (GO:0006783); Biological Process: response to oxidative stress (GO:0006979); Molecular Function: glutamyl-tRNA reductase activity (GO:0008883); Cellular Component: chloroplast (GO:0009507); Biological Process: response to wounding (GO:0009611); Biological Process: response to chitin (GO:0010200); Biological Process: defense response by callose deposition (GO:0052542); |
| 8 | BnaA04g16220D | down | -- |
| 9 | BnaA05g14900D | down | Molecular Function: protein binding (GO:0005515); Cellular Component: nucleus (GO:0005634); Cellular Component: cytosol (GO:0005829); |
| 10 | BnaA05g21710D | up | Cellular Component: nucleus (GO:0005634); Cellular Component: chloroplast envelope (GO:0009941); |
| 11 | BnaA07g00820D | up | Molecular Function: phosphoribosylformylglycinamidine synthase activity (GO:0004642); Molecular Function: ATP binding (GO:0005524); Cellular Component: mitochondrion (GO:0005739); Cellular Component: chloroplast stroma (GO:0009570); Biological Process: microgametogenesis (GO:0055046); |
| 12 | BnaA07g04500D | up | Cellular Component: mitochondrion (GO:0005739); |
| 13 | BnaA07g09660D | up | -- |
| 14 | BnaA07g27870D | up | Molecular Function: zinc ion binding (GO:0008270); |
| 15 | BnaA08g02930D | up | Molecular Function: nucleotide binding (GO:0000166); Biological Process: endonucleolytic cleavage involved in rRNA processing (GO:0000478); Biological Process: RNA methylation (GO:0001510); Molecular Function: nucleic acid binding (GO:0003676); Cellular Component: nucleolus (GO:0005730); Cellular Component: mitochondrion (GO:0005739); Cellular Component: cytosol (GO:0005829); Biological Process: pyrimidine ribonucleotide biosynthetic process (GO:0009220); Biological Process: leaf vascular tissue pattern formation (GO:0010305); Biological Process: cotyledon vascular tissue pattern formation (GO:0010588); Biological Process: root development (GO:0048364); Biological Process: leaf development (GO:0048366); Biological Process: petal vascular tissue pattern formation (GO:0080056); Biological Process: sepal vascular tissue pattern formation (GO:0080057); |
| 16 | BnaA09g06740D | up | Cellular Component: nucleus (GO:0005634); |
| 17 | BnaA09g43250D | up | Cellular Component: mitochondrion (GO:0005739); |
| 18 | BnaA09g44370D | down | Molecular Function: DNA binding (GO:0003677); Molecular Function: chromatin binding (GO:0003682); Molecular Function: sequence-specific DNA binding transcription factor activity (GO:0003700); Cellular Component: nucleus (GO:0005634); Biological Process: regulation of transcription, DNA-templated (GO:0006355); Biological Process: protein targeting to membrane (GO:0006612); Biological Process: response to salt stress (GO:0009651); Biological Process: response to ethylene (GO:0009723); Biological Process: response to auxin (GO:0009733); Biological Process: response to abscisic acid (GO:0009737); Biological Process: response to gibberellin (GO:0009739); Biological Process: response to salicylic acid (GO:0009751); Biological Process: response to jasmonic acid (GO:0009753); Biological Process: positive regulation of flavonoid biosynthetic process (GO:0009963); Biological Process: regulation of plant-type hypersensitive response (GO:0010363); Biological Process: response to cadmium ion (GO:0046686); |
| 19 | BnaA09g44650D | down | Cellular Component: plasma membrane (GO:0005886); Biological Process: proteolysis (GO:0006508); Biological Process: lipid transport (GO:0006869); Molecular Function: peptidase activity (GO:0008233); Molecular Function: lipid binding (GO:0008289); Cellular Component: anchored component of membrane (GO:0031225); |
| 20 | BnaA09g45000D | down | Biological Process: RNA splicing, via endonucleolytic cleavage and ligation (GO:0000394); Molecular Function: DNA binding (GO:0003677); Cellular Component: transcription factor TFIID complex (GO:0005669); Biological Process: DNA-templated transcription, initiation (GO:0006352); Biological Process: transcription from RNA polymerase II promoter (GO:0006366); Biological Process: cytokinin-activated signaling pathway (GO:0009736); Biological Process: jasmonic acid mediated signaling pathway (GO:0009867); Biological Process: regulation of ethylene-activated signaling pathway (GO:0010104); Molecular Function: protein heterodimerization activity (GO:0046982); |
| 21 | BnaA09g45260D | down | Cellular Component: chloroplast (GO:0009507); |
| 22 | BnaA09g45300D | down | Molecular Function: serine-type carboxypeptidase activity (GO:0004185); Cellular Component: extracellular region (GO:0005576); Cellular Component: vacuole (GO:0005773); Biological Process: proteolysis (GO:0006508); |
| 23 | BnaA09g45310D | up | -- |
| 24 | BnaA09g45320D | down | Molecular Function: copper ion binding (GO:0005507); Molecular Function: calmodulin binding (GO:0005516); Molecular Function: ATP binding (GO:0005524); Cellular Component: mitochondrion (GO:0005739); Cellular Component: cytosol (GO:0005829); Biological Process: gluconeogenesis (GO:0006094); Biological Process: glycolytic process (GO:0006096); Biological Process: protein folding (GO:0006457); Biological Process: tryptophan catabolic process (GO:0006569); Biological Process: response to heat (GO:0009408); Biological Process: response to cold (GO:0009409); Cellular Component: chloroplast thylakoid membrane (GO:0009535); Cellular Component: chloroplast stroma (GO:0009570); Biological Process: response to high light intensity (GO:0009644); Biological Process: response to salt stress (GO:0009651); Biological Process: chloroplast organization (GO:0009658); Biological Process: indoleacetic acid biosynthetic process (GO:0009684); Cellular Component: chloroplast envelope (GO:0009941); Biological Process: isopentenyl diphosphate biosynthetic process, methylerythritol 4-phosphate pathway (GO:0019288); Biological Process: cysteine biosynthetic process (GO:0019344); Biological Process: response to endoplasmic reticulum stress (GO:0034976); Biological Process: response to hydrogen peroxide (GO:0042542); Biological Process: response to cadmium ion (GO:0046686); Cellular Component: apoplast (GO:0048046); Biological Process: ovule development (GO:0048481); Molecular Function: chaperone binding (GO:0051087); Biological Process: positive regulation of superoxide dismutase activity (GO:1901671); |
| 25 | BnaA09g45610D | up | Cellular Component: nucleus (GO:0005634); |
| 26 | BnaA09g45890D | down | Cellular Component: plasma membrane (GO:0005886); Biological Process: phosphate ion transport (GO:0006817); Cellular Component: integral component of membrane (GO:0016021); Biological Process: cellular response to phosphate starvation (GO:0016036); |
| 27 | BnaA09g46080D | down | Cellular Component: nucleus (GO:0005634); |
| 28 | BnaA09g47900D | down | Molecular Function: zinc ion binding (GO:0008270); |
| 29 | BnaA09g48320D | down | Molecular Function: structural constituent of ribosome (GO:0003735); Cellular Component: nucleolus (GO:0005730); Biological Process: translation (GO:0006412); Cellular Component: chloroplast (GO:0009507); Cellular Component: cytosolic large ribosomal subunit (GO:0022625); |
| 30 | BnaA09g56410D | down | Biological Process: protein targeting to vacuole (GO:0006623); Cellular Component: membrane (GO:0016020); Biological Process: vesicle-mediated transport (GO:0016192); |
| 31 | BnaA09g56740D | down | -- |
| 32 | BnaA10g00400D | up | Cellular Component: Golgi apparatus (GO:0005794); Molecular Function: transferase activity, transferring glycosyl groups (GO:0016757); |
| 33 | BnaA10g07970D | down | Biological Process: response to stress (GO:0006950); Biological Process: response to abiotic stimulus (GO:0009628); Biological Process: cellular process (GO:0009987); |
| 34 | BnaAnng13790D | up | -- |
| 35 | BnaAnng14640D | up | Molecular Function: GTP binding (GO:0005525); Cellular Component: nucleus (GO:0005634); Cellular Component: cytosol (GO:0005829); |
| 36 | BnaAnng30260D | up | Molecular Function: sequence-specific DNA binding transcription factor activity (GO:0003700); Cellular Component: nucleus (GO:0005634); Biological Process: response to xenobiotic stimulus (GO:0009410); Biological Process: response to ethylene (GO:0009723); Biological Process: hormone-mediated signaling pathway (GO:0009755); Biological Process: endoplasmic reticulum unfolded protein response (GO:0030968); Biological Process: positive regulation of transcription, DNA-templated (GO:0045893); Molecular Function: protein heterodimerization activity (GO:0046982); Biological Process: positive regulation of seed maturation (GO:2000693); |
| 37 | BnaAnng35580D | down | Molecular Function: nucleotide binding (GO:0000166); Biological Process: alternative mRNA splicing, via spliceosome (GO:0000380); Molecular Function: double-stranded DNA binding (GO:0003690); Molecular Function: single-stranded DNA binding (GO:0003697); Molecular Function: mRNA binding (GO:0003729); Molecular Function: protein kinase activity (GO:0004672); Cellular Component: nucleus (GO:0005634); Cellular Component: mitochondrion (GO:0005739); Cellular Component: peroxisome (GO:0005777); Cellular Component: cytosol (GO:0005829); Biological Process: gluconeogenesis (GO:0006094); Biological Process: glycolytic process (GO:0006096); Biological Process: mRNA export from nucleus (GO:0006406); Biological Process: water transport (GO:0006833); Biological Process: hyperosmotic response (GO:0006972); Biological Process: Golgi organization (GO:0007030); Biological Process: response to cold (GO:0009409); Biological Process: response to water deprivation (GO:0009414); Cellular Component: plasmodesma (GO:0009506); Cellular Component: chloroplast (GO:0009507); Biological Process: response to salt stress (GO:0009651); Biological Process: etioplast organization (GO:0009662); Biological Process: lignin biosynthetic process (GO:0009809); Biological Process: response to zinc ion (GO:0010043); Biological Process: regulation of stomatal movement (GO:0010119); Biological Process: response to chitin (GO:0010200); Biological Process: vegetative to reproductive phase transition of meristem (GO:0010228); Biological Process: RNA secondary structure unwinding (GO:0010501); Biological Process: carotenoid biosynthetic process (GO:0016117); Biological Process: brassinosteroid biosynthetic process (GO:0016132); Molecular Function: cinnamoyl-CoA reductase activity (GO:0016621); Biological Process: DNA duplex unwinding (GO:0032508); Biological Process: negative regulation of circadian rhythm (GO:0042754); Molecular Function: protein homodimerization activity (GO:0042803); Biological Process: innate immune response (GO:0045087); Molecular Function: carotenoid isomerase activity (GO:0046608); Biological Process: response to cadmium ion (GO:0046686); Cellular Component: apoplast (GO:0048046); Biological Process: defense response to fungus (GO:0050832); |
| 38 | BnaC01g02500D | up | -- |
| 39 | BnaC01g43270D | up | Molecular Function: magnesium ion binding (GO:0000287); Molecular Function: adenosylmethionine-8-amino-7-oxononanoate transaminase activity (GO:0004015); Molecular Function: dethiobiotin synthase activity (GO:0004141); Molecular Function: ATP binding (GO:0005524); Cellular Component: mitochondrion (GO:0005739); Biological Process: DNA replication initiation (GO:0006270); Biological Process: regulation of DNA replication (GO:0006275); Biological Process: DNA methylation (GO:0006306); Biological Process: cell proliferation (GO:0008283); Biological Process: biotin biosynthetic process (GO:0009102); Molecular Function: pyridoxal phosphate binding (GO:0030170); Biological Process: histone H3-K9 methylation (GO:0051567); Biological Process: regulation of cell cycle (GO:0051726); |
| 40 | BnaC02g06360D | down | Molecular Function: 3-oxo-5-alpha-steroid 4-dehydrogenase activity (GO:0003865); Biological Process: lipid metabolic process (GO:0006629); Cellular Component: chloroplast envelope (GO:0009941); Cellular Component: integral component of membrane (GO:0016021); |
| 41 | BnaC02g06410D | down | Cellular Component: cytoplasm (GO:0005737); Cellular Component: plasmodesma (GO:0009506); Molecular Function: hydrolase activity (GO:0016787); |
| 42 | BnaC02g06440D | down | Cellular Component: cytoplasm (GO:0005737); |
| 43 | BnaC02g06570D | down | Biological Process: maltose metabolic process (GO:0000023); Molecular Function: enzyme inhibitor activity (GO:0004857); Cellular Component: cytosol (GO:0005829); Biological Process: pentose-phosphate shunt (GO:0006098); Biological Process: regulation of carbohydrate metabolic process (GO:0006109); Biological Process: glycerol ether metabolic process (GO:0006662); Molecular Function: enzyme activator activity (GO:0008047); Molecular Function: electron carrier activity (GO:0009055); Cellular Component: chloroplast thylakoid (GO:0009534); Cellular Component: chloroplast stroma (GO:0009570); Molecular Function: protein disulfide oxidoreductase activity (GO:0015035); Biological Process: starch biosynthetic process (GO:0019252); Biological Process: isopentenyl diphosphate biosynthetic process, methylerythritol 4-phosphate pathway (GO:0019288); Biological Process: glucosinolate metabolic process (GO:0019760); Biological Process: electron transport chain (GO:0022900); Biological Process: positive regulation of catalytic activity (GO:0043085); Biological Process: negative regulation of catalytic activity (GO:0043086); Biological Process: cell redox homeostasis (GO:0045454); |
| 44 | BnaC03g09190D | down | -- |
| 45 | BnaC03g19830D | up | -- |
| 46 | BnaC03g24650D | down | Biological Process: cell morphogenesis (GO:0000902); Cellular Component: nucleus (GO:0005634); Cellular Component: Golgi apparatus (GO:0005794); Cellular Component: cytosol (GO:0005829); Cellular Component: plasma membrane (GO:0005886); Biological Process: gluconeogenesis (GO:0006094); Biological Process: glycolytic process (GO:0006096); Biological Process: response to desiccation (GO:0009269); Cellular Component: plasmodesma (GO:0009506); Biological Process: response to salt stress (GO:0009651); Biological Process: embryo development ending in seed dormancy (GO:0009793); Biological Process: cell growth (GO:0016049); Biological Process: cysteine biosynthetic process (GO:0019344); Biological Process: response to cadmium ion (GO:0046686); Biological Process: Golgi vesicle transport (GO:0048193); |
| 47 | BnaC03g57080D | up | Cellular Component: chloroplast (GO:0009507); |
| 48 | BnaC03g63980D | up | Biological Process: RNA splicing, via endonucleolytic cleavage and ligation (GO:0000394); Biological Process: nuclear-transcribed mRNA catabolic process (GO:0000956); Molecular Function: DNA binding (GO:0003677); Molecular Function: DNA-directed RNA polymerase activity (GO:0003899); Cellular Component: DNA-directed RNA polymerase II, core complex (GO:0005665); Cellular Component: cytosol (GO:0005829); Biological Process: transcription from RNA polymerase II promoter (GO:0006366); Cellular Component: plasmodesma (GO:0009506); Biological Process: embryo development ending in seed dormancy (GO:0009793); Molecular Function: ribonucleoside binding (GO:0032549); |
| 49 | BnaC03g65980D | down | Biological Process: fatty acid biosynthetic process (GO:0006633); Cellular Component: membrane (GO:0016020); Molecular Function: transferase activity, transferring acyl groups other than amino-acyl groups (GO:0016747); |
| 50 | BnaC03g76890D | up | Biological Process: protein targeting to vacuole (GO:0006623); Cellular Component: chloroplast (GO:0009507); Cellular Component: membrane (GO:0016020); |
| 51 | BnaC04g10370D | down | Molecular Function: protein kinase activity (GO:0004672); Biological Process: phosphorylation (GO:0016310); |
| 52 | BnaC04g29730D | up | Cellular Component: cytosol (GO:0005829); Biological Process: response to wounding (GO:0009611); Biological Process: response to salicylic acid (GO:0009751); Molecular Function: 12-oxophytodienoate reductase activity (GO:0016629); Biological Process: oxylipin metabolic process (GO:0031407); Biological Process: response to cadmium ion (GO:0046686); |
| 53 | BnaC04g30180D | up | -- |
| 54 | BnaC04g30490D | up | -- |
| 55 | BnaC04g39120D | down | Biological Process: putrescine biosynthetic process (GO:0009446); Molecular Function: N-carbamoylputrescine amidase activity (GO:0050126); |
| 56 | BnaC04g45730D | up | Molecular Function: carboxylic ester hydrolase activity (GO:0004091); Cellular Component: extracellular region (GO:0005576); Biological Process: lipid metabolic process (GO:0006629); Biological Process: vegetative to reproductive phase transition of meristem (GO:0010228); Biological Process: protein desumoylation (GO:0016926); Biological Process: hydrogen peroxide biosynthetic process (GO:0050665); |
| 57 | BnaC05g26860D | down | -- |
| 58 | BnaC05g27130D | down | -- |
| 59 | BnaC05g49350D | down | Molecular Function: 1-phosphatidylinositol 4-kinase activity (GO:0004430); Biological Process: phosphatidylinositol phosphorylation (GO:0046854); |
| 60 | BnaC06g07110D | up | -- |
| 61 | BnaC06g10430D | down | Cellular Component: cytoplasm (GO:0005737); |
| 62 | BnaC06g16950D | up | -- |
| 63 | BnaC06g42000D | up | -- |
| 64 | BnaC07g33980D | up | Molecular Function: protein binding (GO:0005515); Cellular Component: nucleus (GO:0005634); Cellular Component: mitochondrion (GO:0005739); Cellular Component: cytosol (GO:0005829); Biological Process: response to auxin (GO:0009733); Biological Process: systemic acquired resistance, salicylic acid mediated signaling pathway (GO:0009862); Biological Process: defense response to bacterium (GO:0042742); Molecular Function: ADP binding (GO:0043531); |
| 65 | BnaC07g36960D | up | -- |
| 66 | BnaC08g01470D | up | Cellular Component: plasma membrane (GO:0005886); Biological Process: oligopeptide transport (GO:0006857); Molecular Function: kinase activity (GO:0016301); |
| 67 | BnaC08g29060D | down | Biological Process: RNA splicing, via endonucleolytic cleavage and ligation (GO:0000394); Molecular Function: inositol hexakisphosphate binding (GO:0000822); Biological Process: response to molecule of bacterial origin (GO:0002237); Molecular Function: ubiquitin-protein transferase activity (GO:0004842); Cellular Component: nucleus (GO:0005634); Cellular Component: vacuolar membrane (GO:0005774); Biological Process: methionine biosynthetic process (GO:0009086); Biological Process: auxin-activated signaling pathway (GO:0009734); Molecular Function: auxin binding (GO:0010011); Biological Process: stomatal complex morphogenesis (GO:0010103); Biological Process: pollen maturation (GO:0010152); Biological Process: protein ubiquitination (GO:0016567); Biological Process: stamen development (GO:0048443); Biological Process: lateral root development (GO:0048527); Biological Process: photoperiodism, flowering (GO:0048573); Biological Process: cellular response to nitrate (GO:0071249); Biological Process: primary root development (GO:0080022); |
| 68 | BnaC08g35720D | up | Cellular Component: vacuolar proton-transporting V-type ATPase, V0 domain (GO:0000220); Cellular Component: mitochondrion (GO:0005739); Cellular Component: Golgi apparatus (GO:0005794); Biological Process: ATP catabolic process (GO:0006200); Cellular Component: chloroplast (GO:0009507); Molecular Function: hydrogen-translocating pyrophosphatase activity (GO:0009678); Cellular Component: plant-type vacuole membrane (GO:0009705); Molecular Function: hydrogen ion transmembrane transporter activity (GO:0015078); Biological Process: ATP synthesis coupled proton transport (GO:0015986); Biological Process: ATP hydrolysis coupled proton transport (GO:0015991); Molecular Function: ATPase activity (GO:0016887); Biological Process: cellular response to nutrient levels (GO:0031669); Biological Process: sequestering of zinc ion (GO:0032119); Biological Process: vacuolar sequestering (GO:0043181); Molecular Function: nutrient reservoir activity (GO:0045735); Biological Process: vacuolar proton-transporting V-type ATPase complex assembly (GO:0070072); Biological Process: cellular response to salt stress (GO:0071472); |
| 69 | BnaC08g35850D | up | Molecular Function: microtubule motor activity (GO:0003777); Molecular Function: ATP binding (GO:0005524); Cellular Component: cytoplasm (GO:0005737); Cellular Component: kinesin complex (GO:0005871); Cellular Component: microtubule (GO:0005874); Cellular Component: plasma membrane (GO:0005886); Biological Process: microtubule-based movement (GO:0007018); Molecular Function: microtubule binding (GO:0008017); Cellular Component: plasmodesma (GO:0009506); |
| 70 | BnaC08g35880D | down | Cellular Component: cytosol (GO:0005829); Biological Process: ubiquitin-dependent protein catabolic process (GO:0006511); Cellular Component: chloroplast (GO:0009507); |
| 71 | BnaC08g36100D | down | Cellular Component: cytoplasm (GO:0005737); Biological Process: ER to Golgi vesicle-mediated transport (GO:0006888); |
| 72 | BnaC08g36200D | down | Cellular Component: chloroplast (GO:0009507); Biological Process: photorespiration (GO:0009853); |
| 73 | BnaC08g36360D | up | Molecular Function: nucleotide binding (GO:0000166); Molecular Function: catalytic activity (GO:0003824); Cellular Component: mitochondrial respiratory chain complex I (GO:0005747); Biological Process: ubiquitin-dependent protein catabolic process (GO:0006511); Biological Process: response to salt stress (GO:0009651); Biological Process: photorespiration (GO:0009853); Molecular Function: coenzyme binding (GO:0050662); Biological Process: response to misfolded protein (GO:0051788); Biological Process: proteasome core complex assembly (GO:0080129); |
| 74 | BnaC08g37340D | up | Cellular Component: plasma membrane (GO:0005886); Biological Process: proteolysis (GO:0006508); Biological Process: lipid transport (GO:0006869); Molecular Function: peptidase activity (GO:0008233); Molecular Function: lipid binding (GO:0008289); Cellular Component: anchored component of membrane (GO:0031225); |
| 75 | BnaC08g37460D | down | Biological Process: mitotic cell cycle (GO:0000278); Molecular Function: RNA binding (GO:0003723); Molecular Function: polynucleotide adenylyltransferase activity (GO:0004652); Molecular Function: protein binding (GO:0005515); Cellular Component: nucleus (GO:0005634); Biological Process: transcription, DNA-templated (GO:0006351); Biological Process: RNA polyadenylation (GO:0043631); |
| 76 | BnaC08g38200D | down | Cellular Component: nucleus (GO:0005634); Molecular Function: oxidoreductase activity, acting on the CH-CH group of donors, NAD or NADP as acceptor (GO:0016628); |
| 77 | BnaC08g38300D | down | Molecular Function: nucleotide binding (GO:0000166); Biological Process: mRNA splicing, via spliceosome (GO:0000398); Molecular Function: RNA binding (GO:0003723); Molecular Function: protein binding (GO:0005515); Cellular Component: nucleolus (GO:0005730); Biological Process: sugar mediated signaling pathway (GO:0010182); Cellular Component: nuclear speck (GO:0016607); |
| 78 | BnaC08g39020D | up | Cellular Component: cytosol (GO:0005829); Cellular Component: plasmodesma (GO:0009506); |
| 79 | BnaC08g39120D | up | -- |
| 80 | BnaC08g39130D | up | Molecular Function: copper ion binding (GO:0005507); Molecular Function: calmodulin binding (GO:0005516); Molecular Function: ATP binding (GO:0005524); Cellular Component: mitochondrion (GO:0005739); Cellular Component: cytosol (GO:0005829); Biological Process: gluconeogenesis (GO:0006094); Biological Process: glycolytic process (GO:0006096); Biological Process: protein folding (GO:0006457); Biological Process: tryptophan catabolic process (GO:0006569); Biological Process: response to heat (GO:0009408); Biological Process: response to cold (GO:0009409); Cellular Component: chloroplast thylakoid membrane (GO:0009535); Cellular Component: chloroplast stroma (GO:0009570); Biological Process: response to high light intensity (GO:0009644); Biological Process: response to salt stress (GO:0009651); Biological Process: chloroplast organization (GO:0009658); Biological Process: indoleacetic acid biosynthetic process (GO:0009684); Cellular Component: chloroplast envelope (GO:0009941); Biological Process: isopentenyl diphosphate biosynthetic process, methylerythritol 4-phosphate pathway (GO:0019288); Biological Process: cysteine biosynthetic process (GO:0019344); Biological Process: response to endoplasmic reticulum stress (GO:0034976); Biological Process: response to hydrogen peroxide (GO:0042542); Biological Process: response to cadmium ion (GO:0046686); Cellular Component: apoplast (GO:0048046); Biological Process: ovule development (GO:0048481); Molecular Function: chaperone binding (GO:0051087); Biological Process: positive regulation of superoxide dismutase activity (GO:1901671); |
| 81 | BnaC08g39990D | up | Biological Process: MAPK cascade (GO:0000165); Molecular Function: protein serine/threonine kinase activity (GO:0004674); Molecular Function: protein serine/threonine/tyrosine kinase activity (GO:0004712); Molecular Function: ATP binding (GO:0005524); Cellular Component: nucleus (GO:0005634); Cellular Component: cytosol (GO:0005829); Biological Process: protein phosphorylation (GO:0006468); Biological Process: protein targeting to membrane (GO:0006612); Biological Process: response to cold (GO:0009409); Biological Process: response to water deprivation (GO:0009414); Biological Process: response to ethylene (GO:0009723); Biological Process: auxin-activated signaling pathway (GO:0009734); Biological Process: abscisic acid-activated signaling pathway (GO:0009738); Biological Process: brassinosteroid mediated signaling pathway (GO:0009742); Biological Process: systemic acquired resistance, salicylic acid mediated signaling pathway (GO:0009862); Biological Process: jasmonic acid mediated signaling pathway (GO:0009867); Biological Process: regulation of signal transduction (GO:0009966); Biological Process: leaf vascular tissue pattern formation (GO:0010305); Biological Process: regulation of plant-type hypersensitive response (GO:0010363); Biological Process: endoplasmic reticulum unfolded protein response (GO:0030968); Biological Process: negative regulation of defense response (GO:0031348); Biological Process: hyperosmotic salinity response (GO:0042538); Biological Process: negative regulation of programmed cell death (GO:0043069); Biological Process: defense response to fungus (GO:0050832); |
| 82 | BnaC08g40320D | up | Molecular Function: chromatin binding (GO:0003682); Molecular Function: sequence-specific DNA binding transcription factor activity (GO:0003700); Cellular Component: nucleus (GO:0005634); Biological Process: regulation of transcription, DNA-templated (GO:0006355); Biological Process: membrane fusion (GO:0006944); Molecular Function: identical protein binding (GO:0042802); Molecular Function: sequence-specific DNA binding (GO:0043565); Biological Process: Golgi vesicle transport (GO:0048193); |
| 83 | BnaC08g40410D | up | Molecular Function: Ran GTPase activator activity (GO:0005098); Cellular Component: nuclear envelope (GO:0005635); Cellular Component: vacuolar membrane (GO:0005774); Cellular Component: endoplasmic reticulum (GO:0005783); Biological Process: nucleocytoplasmic transport (GO:0006913); Biological Process: toxin catabolic process (GO:0009407); Cellular Component: chloroplast (GO:0009507); Biological Process: photomorphogenesis (GO:0009640); Biological Process: response to salt stress (GO:0009651); Biological Process: cullin deneddylation (GO:0010388); Biological Process: lateral root development (GO:0048527); |
| 84 | BnaC08g40740D | down | Molecular Function: translation initiation factor activity (GO:0003743); Cellular Component: cytoplasm (GO:0005737); |
| 85 | BnaC08g40810D | up | Molecular Function: protein serine/threonine kinase activity (GO:0004674); Biological Process: protein autophosphorylation (GO:0046777); |
| 86 | BnaC08g41180D | down | Molecular Function: DNA binding (GO:0003677); Cellular Component: nucleus (GO:0005634); Molecular Function: zinc ion binding (GO:0008270); |
| 87 | BnaC08g41390D | down | Cellular Component: plant-type vacuole (GO:0000325); Molecular Function: sucrose alpha-glucosidase activity (GO:0004575); Biological Process: carbohydrate metabolic process (GO:0005975); Biological Process: polyamine catabolic process (GO:0006598); Biological Process: calcium ion transport (GO:0006816); Biological Process: iron ion transport (GO:0006826); Biological Process: Golgi organization (GO:0007030); Cellular Component: plant-type cell wall (GO:0009505); Biological Process: response to wounding (GO:0009611); Biological Process: response to bacterium (GO:0009617); Biological Process: response to salt stress (GO:0009651); Biological Process: coumarin biosynthetic process (GO:0009805); Biological Process: cellular response to iron ion starvation (GO:0010106); Biological Process: response to nitrate (GO:0010167); Biological Process: nitrate transport (GO:0015706); Biological Process: brassinosteroid biosynthetic process (GO:0016132); Biological Process: cellular modified amino acid biosynthetic process (GO:0042398); Biological Process: cellular response to gibberellin stimulus (GO:0071370); Biological Process: primary root development (GO:0080022); |
| 88 | BnaC08g41540D | down | Molecular Function: N,N-dimethylaniline monooxygenase activity (GO:0004499); Cellular Component: nucleus (GO:0005634); Biological Process: glucosinolate biosynthetic process (GO:0019761); Molecular Function: flavin adenine dinucleotide binding (GO:0050660); Molecular Function: NADP binding (GO:0050661); Biological Process: oxidation-reduction process (GO:0055114); Molecular Function: 8-methylthiopropyl glucosinolate S-oxygenase activity (GO:0080107); |
| 89 | BnaC08g41720D | down | Molecular Function: aspartic-type endopeptidase activity (GO:0004190); Cellular Component: extracellular region (GO:0005576); Cellular Component: vacuole (GO:0005773); Cellular Component: cytosol (GO:0005829); Biological Process: glycolytic process (GO:0006096); Biological Process: proteolysis (GO:0006508); Biological Process: protein targeting to vacuole (GO:0006623); Biological Process: lipid metabolic process (GO:0006629); Biological Process: water transport (GO:0006833); Biological Process: hyperosmotic response (GO:0006972); Biological Process: Golgi organization (GO:0007030); Biological Process: response to temperature stimulus (GO:0009266); Cellular Component: plasmodesma (GO:0009506); Biological Process: response to salt stress (GO:0009651); Biological Process: response to cadmium ion (GO:0046686); Biological Process: organ development (GO:0048513); |
| 90 | BnaC08g41780D | down | Biological Process: sulfur amino acid metabolic process (GO:0000096); Molecular Function: serine-tRNA ligase activity (GO:0004828); Molecular Function: ATP binding (GO:0005524); Cellular Component: mitochondrion (GO:0005739); Biological Process: rRNA processing (GO:0006364); Biological Process: seryl-tRNA aminoacylation (GO:0006434); Biological Process: mitochondrion organization (GO:0007005); Biological Process: cellular amino acid biosynthetic process (GO:0008652); Biological Process: serine family amino acid metabolic process (GO:0009069); Cellular Component: chloroplast (GO:0009507); Biological Process: embryo development ending in seed dormancy (GO:0009793); Biological Process: chloroplast relocation (GO:0009902); Biological Process: leaf morphogenesis (GO:0009965); Biological Process: thylakoid membrane organization (GO:0010027); Biological Process: photosystem II assembly (GO:0010207); Biological Process: vegetative to reproductive phase transition of meristem (GO:0010228); Biological Process: iron-sulfur cluster assembly (GO:0016226); Biological Process: cell differentiation (GO:0030154); Biological Process: regulation of protein dephosphorylation (GO:0035304); Biological Process: cell wall modification (GO:0042545); Biological Process: transcription from plastid promoter (GO:0042793); Biological Process: positive regulation of transcription, DNA-templated (GO:0045893); Biological Process: ovule development (GO:0048481); |
| 91 | BnaC08g42080D | down | Cellular Component: plasma membrane (GO:0005886); Molecular Function: transferase activity, transferring phosphorus-containing groups (GO:0016772); |
| 92 | BnaC08g42280D | down | Biological Process: telomere maintenance (GO:0000723); Biological Process: double-strand break repair via homologous recombination (GO:0000724); Molecular Function: nucleic acid binding (GO:0003676); Molecular Function: ATP binding (GO:0005524); Cellular Component: nucleus (GO:0005634); Biological Process: DNA replication (GO:0006260); Cellular Component: plasmodesma (GO:0009506); Biological Process: vegetative to reproductive phase transition of meristem (GO:0010228); Molecular Function: ATP-dependent 3'-5' DNA helicase activity (GO:0043140); Biological Process: cellular response to cold (GO:0070417); Biological Process: cellular response to abscisic acid stimulus (GO:0071215); |
| 93 | BnaC08g42450D | down | Biological Process: response to molecule of bacterial origin (GO:0002237); Molecular Function: protein serine/threonine kinase activity (GO:0004674); Molecular Function: ATP binding (GO:0005524); Cellular Component: plasma membrane (GO:0005886); Biological Process: N-terminal protein myristoylation (GO:0006499); Biological Process: protein targeting to membrane (GO:0006612); Biological Process: membrane fusion (GO:0006944); Biological Process: response to oxidative stress (GO:0006979); Biological Process: transmembrane receptor protein tyrosine kinase signaling pathway (GO:0007169); Biological Process: systemic acquired resistance (GO:0009627); Biological Process: seed germination (GO:0009845); Biological Process: stomatal complex morphogenesis (GO:0010103); Biological Process: regulation of plant-type hypersensitive response (GO:0010363); Cellular Component: integral component of membrane (GO:0016021); Biological Process: negative regulation of programmed cell death (GO:0043069); Biological Process: protein autophosphorylation (GO:0046777); Biological Process: stamen development (GO:0048443); Cellular Component: micropyle (GO:0070825); |
| 94 | BnaC08g49500D | up | Cellular Component: chloroplast (GO:0009507); Biological Process: systemic acquired resistance (GO:0009627); Biological Process: regulation of defense response (GO:0031347); |
| 95 | BnaC08g49610D | down | Molecular Function: protein binding (GO:0005515); Molecular Function: ATP binding (GO:0005524); Cellular Component: mitochondrion (GO:0005739); Biological Process: starch catabolic process (GO:0005983); Biological Process: circadian rhythm (GO:0007623); Cellular Component: chloroplast stroma (GO:0009570); Biological Process: response to symbiotic fungus (GO:0009610); Biological Process: cold acclimation (GO:0009631); Cellular Component: chloroplast envelope (GO:0009941); Biological Process: phosphorylation (GO:0016310); Biological Process: starch biosynthetic process (GO:0019252); Molecular Function: alpha-glucan, water dikinase activity (GO:0050521); |
| 96 | BnaC09g05590D | up | Molecular Function: pectinesterase activity (GO:0030599); Biological Process: negative regulation of catalytic activity (GO:0043086); Molecular Function: pectinesterase inhibitor activity (GO:0046910); |
| 97 | BnaC09g05960D | up | Molecular Function: DNA binding (GO:0003677); Cellular Component: nucleus (GO:0005634); |
| 98 | BnaC09g06110D | up | Cellular Component: extracellular region (GO:0005576); Cellular Component: endoplasmic reticulum (GO:0005783); Cellular Component: chloroplast (GO:0009507); Biological Process: unidimensional cell growth (GO:0009826); Molecular Function: glucosidase activity (GO:0015926); Biological Process: cellulose biosynthetic process (GO:0030244); Molecular Function: carbohydrate binding (GO:0030246); Biological Process: defense response to bacterium (GO:0042742); Biological Process: response to cadmium ion (GO:0046686); Biological Process: Golgi vesicle transport (GO:0048193); |
| 99 | BnaC09g06220D | up | Cellular Component: plasma membrane (GO:0005886); Cellular Component: chloroplast (GO:0009507); |
| 100 | BnaC09g06260D | up | Cellular Component: nucleus (GO:0005634); |
| 101 | BnaCnng17490D | up | -- |
| 102 | BnaCnng24040D | up | Molecular Function: protein binding (GO:0005515); Cellular Component: cytosol (GO:0005829); Biological Process: glycolytic process (GO:0006096); Biological Process: tricarboxylic acid cycle (GO:0006099); Biological Process: iron ion transport (GO:0006826); Biological Process: water transport (GO:0006833); Biological Process: hyperosmotic response (GO:0006972); Biological Process: Golgi organization (GO:0007030); Molecular Function: phosphoenolpyruvate carboxylase activity (GO:0008964); Biological Process: response to temperature stimulus (GO:0009266); Biological Process: response to salt stress (GO:0009651); Biological Process: response to sucrose (GO:0009744); Biological Process: response to glucose (GO:0009749); Biological Process: response to fructose (GO:0009750); Biological Process: cellular response to iron ion starvation (GO:0010106); Biological Process: response to nitrate (GO:0010167); Biological Process: nitrate transport (GO:0015706); Biological Process: carbon fixation (GO:0015977); Biological Process: cellular response to phosphate starvation (GO:0016036); Biological Process: response to cadmium ion (GO:0046686); Cellular Component: apoplast (GO:0048046); Biological Process: protein tetramerization (GO:0051262); |
| 103 | BnaCnng36050D | up | -- |
| 104 | BnaCnng49710D | down | -- |
| 105 | BnaCnng68410D | up | Biological Process: transcription, DNA-templated (GO:0006351); Biological Process: RNA processing (GO:0006396); Biological Process: translational initiation (GO:0006413); Biological Process: post-embryonic development (GO:0009791); Biological Process: posttranscriptional regulation of gene expression (GO:0010608); Cellular Component: macromolecular complex (GO:0032991); Biological Process: macromolecule localization (GO:0033036); Cellular Component: cytoplasmic part (GO:0044444); Biological Process: single-organism developmental process (GO:0044767); Biological Process: reproductive structure development (GO:0048608); Biological Process: cellular response to organic substance (GO:0071310); Biological Process: regulation of primary metabolic process (GO:0080090); Biological Process: regulation of cellular macromolecule biosynthetic process (GO:2000112); |
| 106 | BnaCnng75420D | up | Cellular Component: vacuolar proton-transporting V-type ATPase, V0 domain (GO:0000220); Cellular Component: mitochondrion (GO:0005739); Cellular Component: Golgi apparatus (GO:0005794); Biological Process: ATP catabolic process (GO:0006200); Cellular Component: chloroplast (GO:0009507); Molecular Function: hydrogen-translocating pyrophosphatase activity (GO:0009678); Cellular Component: plant-type vacuole membrane (GO:0009705); Molecular Function: hydrogen ion transmembrane transporter activity (GO:0015078); Biological Process: ATP synthesis coupled proton transport (GO:0015986); Biological Process: ATP hydrolysis coupled proton transport (GO:0015991); Molecular Function: ATPase activity (GO:0016887); Biological Process: cellular response to nutrient levels (GO:0031669); Biological Process: sequestering of zinc ion (GO:0032119); Biological Process: vacuolar sequestering (GO:0043181); Molecular Function: nutrient reservoir activity (GO:0045735); Biological Process: vacuolar proton-transporting V-type ATPase complex assembly (GO:0070072); Biological Process: cellular response to salt stress (GO:0071472); |
| 107 | Cole_newGene_1596 | down | -- |
| 108 | Cole_newGene_1717 | down | Molecular Function: binding (GO:0005488); |
| 109 | Cole_newGene_1891 | up | -- |
| 110 | Cole_newGene_1939 | up | -- |
| 111 | Cole_newGene_1983 | down | Cellular Component: mitochondrion (GO:0005739); Cellular Component: chloroplast stroma (GO:0009570); Biological Process: mRNA modification (GO:0016556); Molecular Function: carbon-nitrogen ligase activity, with glutamine as amido-N-donor (GO:0016884); |
| 112 | Cole_newGene_1984 | down | Cellular Component: plant-type vacuole (GO:0000325); Cellular Component: vacuolar membrane (GO:0005774); Cellular Component: chloroplast (GO:0009507); Biological Process: response to salt stress (GO:0009651); Biological Process: proton transport (GO:0015992); Biological Process: cellular component organization (GO:0016043); Molecular Function: pyrophosphatase activity (GO:0016462); Molecular Function: transmembrane transporter activity (GO:0022857); Biological Process: ion transmembrane transport (GO:0034220); Biological Process: ATP metabolic process (GO:0046034); Biological Process: maintenance of location (GO:0051235); Biological Process: cellular localization (GO:0051641); Biological Process: cellular response to stimulus (GO:0051716); |
| 113 | Cole_newGene_1990 | down | -- |
| 114 | Cole_newGene_2071 | down | Molecular Function: sequence-specific DNA binding transcription factor activity (GO:0003700); Cellular Component: nucleus (GO:0005634); Biological Process: regulation of transcription, DNA-templated (GO:0006355); Molecular Function: protein dimerization activity (GO:0046983); |
| 115 | Cole_newGene_2073 | up | Cellular Component: intracellular membrane-bounded organelle (GO:0043231); Cellular Component: cytoplasmic part (GO:0044444); |
| 116 | Cole_newGene_2243 | down | Cellular Component: mitochondrion (GO:0005739); Cellular Component: Golgi apparatus (GO:0005794); Cellular Component: plasma membrane (GO:0005886); Biological Process: response to stress (GO:0006950); Cellular Component: integral component of endoplasmic reticulum membrane (GO:0030176); Biological Process: single-organism cellular process (GO:0044763); |
| 117 | Cole_newGene_2682 | up | -- |
| 118 | Cole_newGene_269 | up | -- |
| 119 | Cole_newGene_2756 | up | -- |
| 120 | Cole_newGene_3294 | up | -- |
| 121 | Cole_newGene_3682 | down | -- |
| 122 | Cole_newGene_3766 | down | -- |
| 123 | Cole_newGene_4151 | down | -- |
| 124 | Cole_newGene_4761 | up | -- |
| 125 | Cole_newGene_5614 | up | -- |
| 126 | Cole_newGene_6035 | up | Molecular Function: hydrolase activity, acting on ester bonds (GO:0016788); |
| 127 | Cole_newGene_6107 | up | -- |
| 128 | Cole_newGene_6687 | up | Cellular Component: cell wall (GO:0005618); Cellular Component: vacuole (GO:0005773); Cellular Component: endoplasmic reticulum (GO:0005783); Cellular Component: plasma membrane (GO:0005886); Cellular Component: plasmodesma (GO:0009506); Molecular Function: transmembrane transporter activity (GO:0022857); Biological Process: transmembrane transport (GO:0055085); |
| 129 | Cole_newGene_959 | up | Molecular Function: endopeptidase activity (GO:0004175); Molecular Function: ubiquitin-protein transferase activity (GO:0004842); Molecular Function: protein binding (GO:0005515); Cellular Component: extracellular region (GO:0005576); Cellular Component: endoplasmic reticulum (GO:0005783); Biological Process: postreplication repair (GO:0006301); Biological Process: proteolysis (GO:0006508); Biological Process: response to auxin (GO:0009733); Biological Process: embryo development (GO:0009790); Biological Process: protein processing (GO:0016485); Biological Process: protein ubiquitination (GO:0016567); Molecular Function: NEDD8 transferase activity (GO:0019788); Biological Process: cellular protein localization (GO:0034613); Biological Process: CAAX-box protein maturation (GO:0080120); |
