# Supplementary material for: Identification of genomic regions associated with multi-silique trait in Brassica napus
Source: BMC Genomics. 2019 Apr 23;20:304. doi: 10.1186/s12864-019-5675-4 (PMC6480887; doi:10.1186/s12864-019-5675-4)
Supplement: Supplementary file 12 — Figure S2. Stability of the multi-silique trait under one environment and variation of this trait across different environments. (DOCX 1840 kb) [file 12864_2019_5675_MOESM12_ESM.docx]

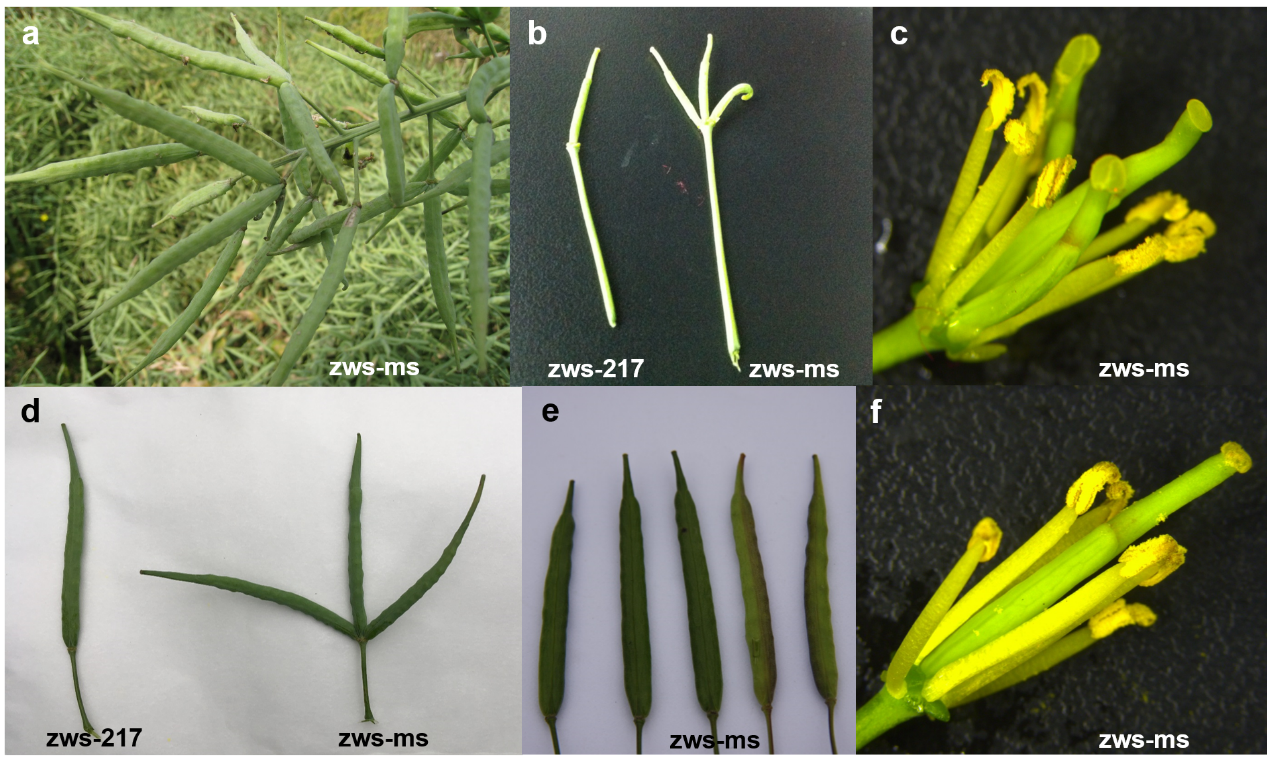


Additional file12: Fig S2. Stability of the multi-silique trait under one environment and variation of this trait across different environments. The zws-ms plants showed stable multi-silique trait in Xindu in continuous years; when it was grown in Ma’erkang, it turned into normal flower/silique.

a): Mature pods: multi-siliques from zws-ms observed in Xindu, 2015.

b): Young pods: multi-siliques from zws-ms compared with normal silique from zws-217 in Xindu, 2016.

c): A floral organ from zws-ms, with petals and calyces peeled off, showing multiple pistils and stamens in Xindu, 2017.

d): Mature pods: multi-siliques from zws-ms compared with normal silique from zws-217 in 2018 (also showed in Fig 2).

e): zws-ms developed single-silique in Ma’erkang, 2016.

f): A floral organ from zws-ms grown in Ma’erkang, with petals and calyces peeled off, showing normal structure, 2017.
