# Supplementary material for: Identification of genomic regions associated with multi-silique trait in Brassica napus
Source: BMC Genomics. 2019 Apr 23;20:304. doi: 10.1186/s12864-019-5675-4 (PMC6480887; doi:10.1186/s12864-019-5675-4)
Supplement: Supplementary file 13 — Table S11. Primers used for qPCR. (DOCX 15 kb) [file 12864_2019_5675_MOESM13_ESM.docx]

Additional file 13: Table S11. Primers used for qPCR

| Primer | Sequence |
| --- | --- |
| Bna-actin-7-forward | 5’-CTGGAATTGCTGACCGTATGAG-3’ |
| Bna-actin-7-reverse | 5’-GCCAAGATGGATCCTCCAATC-3’ |
| BnaA09g45320D-forward | 5’-TAGAATTCACACTCACCACCGAT-3’ |
| BnaA09g45320D-reverse | 5’-GGTTTTAGCAGGCTGGATGAC-3’ |
| BnaC08g41780D-forward | 5’-AGAGGCAAGTGGTTCAAAGTTC-3’ |
| BnaC08g41780D-reverse | 5’-CCTTCTTCATGACTTCTGATAACG-3’ |
| BnaC08g41720D-forward | 5’-CAACGATCTATGCGAGCGTC-3’ |
| BnaC08g41720D-reverse | 5’-CAGTGGGCATGGTCGAGAG-3’ |
| BnaA09g47900D-forward | 5’-GCCTCGTTCTACACTGCTCTTC-3’ |
| BnaA09g47900D-reverse | 5’-CGCAGGGAAACAATCCAAG-3’ |
| BnaC08g40740D-forward | 5’-TCTTGAACCACGAAAGGCTGT-3’ |
| BnaC08g40740D-reverse | 5’-TTGCTCTCTAGGTCTTGCTGCT-3’ |
| BnaC08g42080D-forward | 5’-CCCATCTTCCTAACCTCGCT-3’ |
| BnaC08g42080D-reverse | 5’-ATGCTGGGAGTGAACAGACAAG-3’ |
| BnaA09g45890D-forward | 5’-GCTTAACAAACAGATGGACGCT-3’ |
| BnaA09g45890D-reverse | 5’-ATCTCCACCGCACGTTCATC-3’ |
| BnaC08g40410D-forward | 5’-GAAGCTTGATCTGAGGGATAACAT-3’ |
| BnaC08g40410D-reverse | 5’-AGCTCCGTCAAGTGTTTGTAGC-3’ |
| BnaA09g45310D-forward | 5’-TACGCTGGATTCTACTGCTTCAC-3’ |
| BnaA09g45310D-reverse | 5’-TGCATAAAACTGATCTCCCCTG-3’ |
| BnaC08g39120D-forward | 5’-CTCTTACGCTGGATTCTACTGCT-3’ |
| BnaC08g39120D-reverse | 5’-GCAGGGTAAGATGCATAGAACTG-3’ |
